# A multi-stakeholder perspective on barriers to the adoption of personalized prevention in European healthcare systems

**DOI:** 10.3389/fpubh.2026.1817705

**Published:** 2026-06-24

**Authors:** Alexandra Costa, Maria Luís Cardoso, Adriana Reimão Carvalho, Tommaso Osti, Sara Farina, Abdelrahman Taha, Cosimo Savoia, Luigi Russo, Alessandra Maio, Nicolò Scarsi, Evelina Flodkvist, Alexandra Gyllenberg, Stefania Boccia, Astrid Moura Vicente

**Affiliations:** 1 Department of Health Promotion and NCDs Prevention, National Health of Institute Doctor Ricardo Jorge, Lisbon, Portugal; 2 Faculty of Sciences, BioISI–Biosystems and Integrative Sciences Institute, University of Lisbon, Lisbon, Portugal; 3 Section of Hygiene, Department of Life Sciences and Public Health, Università Cattolica del Sacro Cuore, Rome, Italy; 4 Department of Medicine and Surgery, University of Perugia, Perugia, Italy; 5 Department of Global Public Health, Karolinska Institutet, Stockholm, Sweden; 6 Department of Woman and Child Health and Public Health, Fondazione Policlinico Universitario A. Gemelli IRCCS, Rome, Italy

**Keywords:** clinical practice, genetics, interventions, non-communicable diseases, personalized prevention

## Abstract

**Introduction:**

Personalized prevention is an important component of personalized medicine, tailoring interventions to the biological, behavioral, sociocultural and environmental characteristics of individuals for the prevention of disease onset, progression and recurrence. Despite its potential, personalized preventive interventions (PPI) remain less commonly implemented in clinical practice. This study explored the main barriers to a broader adoption of PPI in European healthcare systems.

**Methods:**

A multi-stakeholder consultation involving citizens/patients, health professionals, researchers, and policymakers was conducted using a sequential mixed-methods approach. In the first phase, semi-structured interviews with key informants representing different stakeholder groups were conducted. The thematic analysis of interview findings, complemented by insights from a review of the literature, informed the development of an online survey implemented in the second phase of the study.

**Results:**

Twenty-six interviews and 270 complete surveys were analyzed. Stakeholders identified barriers across three main domains: (1) healthcare systems, (2) implementation, and (3) awareness, education, and literacy. Key barriers associated with limited PPI adoption included the predominant focus of health strategies on treatment over prevention, unresolved ethical, legal, and social issues (ELSI), limited awareness and knowledge of personalized prevention among both professionals and citizens, and the lack of appropriate cost–benefit evaluation models.

**Conclusion:**

Findings highlight interconnected barriers that may impact a broader PPI adoption across healthcare system, governance, implementation, and awareness-related domains. Continued stakeholder dialogue and engagement, alongside efforts to address awareness and governance-related challenges, may support the broader integration of PPI into European healthcare systems.

## Introduction

Personalized Medicine (PM) refers to a healthcare approach that aims to tailor diagnostic, preventive, and therapeutic strategies according to individual biological, clinical, environmental, behavioral, socio-economic, and cultural characteristics (1). Moving from the traditional “one-size-fits-all” approach toward a patient-centered healthcare PM enables individualized approaches to stratify populations to identify who benefits most from a given intervention with the goal to reduce the burden of disease and improve quality of life (2, 3). The integration of this concept with classical paradigms of prevention fosters a new approach, which is called personalized prevention (4).

Personalized Prevention (PP) represents the preventive dimension of PM and focuses specifically on identifying individual disease risks and tailoring preventive strategies accordingly (5). PP aims to support earlier and more targeted preventive care by integrating personal health information, including genetic predisposition, environmental exposures, and behavioral risk factors, into risk stratification and prevention planning (5–10). PP also acknowledges that effective preventive interventions should account not only for individual biological and behavioral risk factors, but also for the broader social, environmental, and psychosocial dimensions that may influence health outcomes and individual wellbeing, particularly in the context of chronic and complex diseases (8).

Although PM and PP are conceptually interconnected and partially overlapping, in this study PM is used to refer to the broader patient-centered healthcare paradigm, whereas PP specifically refers to its prevention-focused dimension (3, 5, 7). Therefore, the term personalized preventive intervention (PPI) is adopted to refer to the concrete person-centered preventive actions, clinical strategies and interventions developed based on PP principles.

PPI can be implemented across the various levels of prevention to improve health outcomes and quality of life (10). At the primary level, PPI include risk prediction models to support tailored lifestyle interventions delaying disease onset (9, 10). At the secondary level, PPI uses risk-stratified screening approaches based on genetic and other biomarkers to identify individuals at higher risk and enable earlier intervention (9, 10). PPI at the tertiary level include pharmacogenetic testing, targeted therapies, and other strategies to improve disease management, reduce complications and improve quality of life (10).

Despite growing evidence of the potential benefits of PPI, their adoption within healthcare systems remains more limited than the adoption of personalized approaches focused on diagnosis and treatment and varies across countries and healthcare systems (11, 12). This asymmetric adoption may contribute to widening existing health inequalities, limiting equitable access to PPI (9).

Several barriers affecting the implementation of PPI have been identified in previous studies, as reviewed by Scarsi et al. (13) However, current evidence remains fragmented, as it is often focused either on PM broadly or on isolated implementation challenges within specific healthcare settings or stakeholder groups. Furthermore, the integration of the perspectives of multiple stakeholder groups regarding the main barriers hampering a wider adoption of PPI in healthcare systems has not been sufficiently explored.

To address this gap, we conducted a two-step consultation process, consisting of interviews followed by an online survey informed by the qualitative findings. We collected perspectives from four key stakeholder groups: citizens and patients, health professionals, researchers, and policymakers. The inclusion of these stakeholder groups was endorsed by the multidimensional nature of personalized prevention implementation, which requires consideration of end-user perspectives, healthcare workforce preparedness, organizational and governance dimensions, as well as ethical, legal, and financial factors (12). The engagement of these stakeholder groups is also critical across the three domains that are central to the implementation of PPI: Research, Care, and Governance (14). While researchers contribute primarily to the generation of scientific evidence, technological innovation, and the development and validation of PPI, healthcare professionals play a central role in the delivery and integration of PPI into clinical settings. Policymakers contribute to governance, regulation, and resource allocation, whereas citizens and patients are essential to understanding end-users’ acceptability, preferences and access to PPI. These stakeholder groups contribute in interconnected and complementary ways throughout processes of research, healthcare delivery, and governance. The successful adoption of PPI therefore requires balancing the expectations, priorities, and concerns of all actors involved in the loop of implementation.

By integrating perspectives from different stakeholder groups, this study contributes to a comprehensive and integrated understanding of perceived barriers hindering the adoption of PPI and to outline a set of recommendations aiming to foster an effective and equitable adoption of PPI within European healthcare systems. The study was conducted within the framework of the EU-funded project A PeRsOnalised Prevention roadmap for the future HEalThcare (PROPHET),^1^ which aims to support the implementation of innovative, sustainable, and effective personalized programs for the prevention of common chronic diseases (5).

## Materials and methods

The study protocol was approved by Ethical Committees from the National Institute of Health Doctor Ricardo Jorge (INSA, Portugal) and the Catholic University of the Sacred Heart (UCSC, Italy) (approval number 5658).

### Study design

This study adopted a sequential mixed-methods design consisting of two consecutive phases: semi-structured interviews followed by an online survey. The interview phase aimed to explore stakeholders’ perceptions regarding barriers for the implementation of personalized preventive interventions (PPI) within healthcare systems. Interview guides were developed based on the existing literature on personalized prevention and healthcare innovation, while maintaining sufficient flexibility to allow the exploration of emerging topics during the interviews.

The findings from the thematic analysis of the interviews were subsequently integrated with insights from a targeted review of the literature. This combined analytical process informed the development and structuring of the survey instrument. Accordingly, the survey was designed to further explore, validate, and prioritize barriers identified during the qualitative interview phase, while enabling the inclusion of perspectives from a broader multi-stakeholder sample.

### Interviews

#### Interview guide development

Four semi-structured interview guides were developed targeting each of the stakeholder groups included in the study: citizens/patients, health professionals, researchers, and policymakers. Although tailored to the specific roles and perspectives of each stakeholder group regarding the implementation of personalized preventive interventions (PPI), all interview guides followed a common thematic structure informed by the literature on PP and healthcare innovation and the expertise of the research team.

The interviews explored participants’ perceptions regarding barriers to PPI, including challenges related to investment and funding for prevention services and research, equity and access dimensions in the implementation of PPI, the quality and availability of evidence supporting PPI adoption, healthcare professionals’ knowledge and preparedness, and citizens’ and patients’ awareness and acceptance of personalized prevention approaches. Example questions included: “What are the main barriers to the implementation of personalized prevention in healthcare systems?”; “How adequate is the current evidence base supporting personalized preventive interventions?”; and “What challenges may affect equitable access to personalized prevention approaches?.” While predefined thematic areas guided the interviews, sufficient flexibility was maintained to allow participants to elaborate on issues relevant to their specific stakeholder perspective (Supplemental Material Interview Guides 1–4).

#### Recruitment

Participants were purposively identified to ensure representation across the four stakeholder groups included in the study: citizens/patients, health professionals, researchers, and policymakers. Eligible participants were individuals with professional expertise, policy experience, research involvement, or lived experience relevant to personalized prevention and healthcare systems, including representatives from patient and citizen organizations involved in European and national networks, such as the PROPHET Stakeholder Forum. Given the exploratory and descriptive nature of the study, participant selection aimed to maximize diversity of perspectives and expertise rather than statistical representativeness.

Interviews were conducted between April and June 2023, in English or in the participant national language (Portuguese, Italian or Spanish). A total of 26 participants took part in the interviews, including 11 health professionals, 6 researchers, 5 policymakers, and 4 patient representatives. An equal gender distribution was achieved, with 13 female and 13 male participants. Participants were affiliated with different European organizations across several countries and contributed their individual perspectives including professional, institutional, advocacy, and experiential perspectives relevant to the implementation of PPI (see Supplementary Table 1). Individual interviews lasted between 45 and 60 min.

#### Content analysis and data synthesis

Interviews were conducted online via Microsoft Teams, audio-recorded with participants’ consent, and transcribed verbatim for analysis. Members of the INSA and UCSC research teams conducted the interviews and were responsible for the transcription and quality verification of the interviews they conducted. As some interviews were conducted in participants’ native languages, all final transcripts used for content analysis were translated into English to ensure consistency in the analytical process. Microsoft Excel was used to support thematic coding, data organization, and synthesis.

Qualitative content analysis was conducted using reflexive thematic analysis following Braun and Clarke (15), aiming to identify and interpret recurring patterns across interviews. One researcher read all transcripts multiple times and produced analytical memos documenting emerging interpretations, relevant quotations, and coding rationales. Initial coding was conducted inductively using an open coding approach. Although the interview guides included predefined thematic areas informed by the literature, the semi-structured format allowed participants to introduce additional perspectives and emerging issues, supporting an inductive and reflexive analytical approach. Themes and sub-themes were subsequently developed and iteratively reviewed against coded extracts and the full dataset by two authors to ensure coherence and consistency with participants’ accounts. Differences in coding interpretation and thematic organization were resolved through discussion and consensus between the two authors. Themes were subsequently organized into five overarching domains to support the interpretation and presentation of barriers affecting the implementation of PPI. These domains served as an analytical framework for organizing related themes and sub-themes within the overall implementation context. The final thematic structure was subsequently reviewed and discussed with all co-authors to ensure the consistency and credibility of the analysis and served as the basis for both the interpretation of qualitative findings and the development of the survey instrument.

### Survey

A cross-sectional online survey was developed based on interviews outcomes, to collect perspectives from a wider audience of each stakeholder group on perceived barriers to the adoption of PPI. More specifically, the survey aimed to further explore barriers identified during the interviews, including challenges related to investment in prevention, availability of evidence supporting PPI, equity and access issues, healthcare professionals’ preparedness, citizens’ awareness and acceptance, governance-related barriers, and challenges related to intersectoral collaboration affecting the implementation of PPI. The survey was developed in English and administered through the REDCap platform, with an estimated time of 15 min to be completed.

#### Survey instrument

Survey items were developed by the research team based on the thematic analysis of the interviews and complemented with findings from the literature review (see Supplementary Survey). To support the development and organization of the survey instrument, a matrix-based approach was used to map survey questions against the themes and related domains emerging from the interviews, ensuring consistency between the qualitative findings from the interviews and the survey instrument, as well as broad coverage of the main implementation dimensions identified during the qualitative phase. The questionnaire items covered barriers related to citizens’ awareness and acceptance, healthcare professionals’ preparedness, equity and access issues, investment in prevention, evidence and knowledge translation gaps, governance-related barriers, and challenges associated with intersectoral collaboration.

The level of agreement with given statements was scored on a 6-point Likert scale (from “Strongly disagree” to “Strongly agree”).

Prior to dissemination, the survey instrument was reviewed internally by all members of the research team to assess clarity and consistency with the study objectives.

#### Recruitment and dissemination strategy

Invitations to participate and survey links were disseminated through multiple stakeholder and professional networks using a broad snowball approach. Members of the PROPHET Consortium were asked to circulate the survey through their institutional channels, professional networks, and stakeholder contacts. In addition, members of the research team disseminated the survey through national and international networks, including scientific societies, healthcare organizations, and patient and citizen associations. Given the exploratory and multi-stakeholder nature of the study, participation was open to individuals with professional, research, policy, or lived experience relevant to personalized prevention. Participants were asked to self-identify as belonging to one of the four stakeholder groups included in the study and to report their familiarity with personalized preventive interventions. Sociodemographic and other stakeholder information was collected to characterize the diversity of perspectives represented in the survey sample. Agreement to participate was requested in the introductory section. Data collection took place between June 2023 and March 2024.

#### Data analysis

Descriptive statistics were used to analyze sociodemographic data. Likert-scale answers were treated as categorical data with frequencies represented by bar charts. To facilitate the interpretation of the findings, perceptions of barriers were grouped in two main categories: *disagreement* (combining Strongly disagree and Disagree answers) vs. *agreement* (combining Strongly agree and Agree answers). Neutral options (Neither agree or disagree and Do not know) are also presented. The adopted analytical approach was aligned with the exploratory and descriptive nature of the study and aimed to identify general patterns of perception across stakeholder groups rather than establish inferential comparisons or population-level generalizations.

## Results

### Interviews with stakeholders

The stakeholders perceived barriers to a wider adoption of PPI were systematized in 5 main domains: Healthcare systems, Research, Implementation, Awareness, education and literacy, and Personal attitudes. Overall, 13 themes with 28 associated sub-themes captured the stakeholders’ perceived barriers to the adoption of PPI, categorized into the five main levels, with illustrative quotes (Table 1). The barriers identified through the thematic analysis are presented below, organized into five overarching domains encompassing the main themes and sub-themes.

**Table 1:** Main barriers to the adoption of personalized preventive interventions according to the thematic analysis of stakeholders’ interviews.

Domain	Themes	Sub-themes and codes	Representative quotes
Healthcare system	Health strategy	Focus on disease treatment not prevention Lack of a sense of political attention and priority for prevention Generalized resistance to prevention Investment focus on treatment Limited and low visibility of personalized preventive strategies Tertiary prevention focus Lack of strategy for personalized prevention Limited support from supra-national institutions Lack of assessment of medium/long term impact of current options Lack of an overarching framework for prevention including other determinants of health and health in all policies approach Siloed perspective of health care expenditure Lack of clear roles and responsibilities of all stakeholders involved in personalized prevention Lack of coordination between different actors Insufficient investment Insufficient funding for implementation of personalized prevention strategies Competition for scarce resources Competing priorities and demands for public investment Inadequate economic models Inadequate economic models for personalized prevention Lack of a business model for prevention Insufficient research on economic models for personalized prevention Lack of demonstration of cost benefits of personalized prevention Insufficient awareness of benefits due to inadequate business models Scepticism on the cost-effectiveness of personalized prevention strategies Personalized preventive strategies are perceived as costly and difficult to implementation in a short-time frame	“(…) most of the healthcare systems are simply not oriented at prevention. They are oriented at curing or first diagnosing and then curing disease. (…) the education of people is pointed at diagnosing and curing. The reimbursement system is pointed at diagnosing.” [R1] “(…) the bet is based more on treatment than on prevention. With an increasing lack of resources, it is barely possible to provide adequate treatments at the ideal time for patients (…)” [CP2] “(…) implementation (…) is very fragmented, you have different authorities, organizations, (…) different actors, but it is not in a really coordinated order. There it is not one big strategy yet.” [PM2] “(…) fail to fully assess the medium- and long-term impact of choices that weigh today but could save a lot tomorrow, this is due to a siloed perspective of health care spending.” [CP4] “We need to shift the perception, particularly among politicians, policymakers, and the general public, that personalized prevention is a burden on the healthcare (…) it is essential to reframe personalized prevention as an investment rather than a cost. This will help generate more support and funding for public health initiatives, including research, thereby calling for increased investments in these areas.” [PM5] “(…) an issue to overcome is related to the financing. (…) should we argue a shift of money from care to personalized prevention (…)? And should we ask money from other sectors to pay for personalized prevention? That is, indeed, an issue to see what strategically would work best.” [PM2] “(…) it is not seen by everyone that prevention pays off, so we need to be better at demonstrating the costs and the calculation of the return on investment. And that health outcomes will improve by investing in prevention.” [PM2]
	Inequities in access	Equity asymmetries Existing access asymmetries across regions Need to travel to have access to personalized prevention strategies Access to personalized prevention strategies is often not reimbursed Patient and citizens out-of-pocket expenses are individually supported costs Effect of socioeconomic status Impact of the political determinants of health in access and health outcomes	(…) personalized prevention is very dependent on access to a set of diagnostics that are expensive and that we are in no way ensuring universal and equitable access to. (…).” [PM1] “(…) accessibility and affordability to personalized prevention programs (…) can be limited by several barriers, such as geographical, financial, logistical (…).” [PM5] “(…) there may also be barriers in the difficulty of accessing screening. Sometimes people may have to travel a lot to be screened, and this is not always easy, especially for older people.” [CP2] “(…) there are positive examples of personalized prevention strategies, but these are limited and, more importantly, not very visible (…) strategies often appear fragmented in regionalized health systems, which creates a lot of uncertainty for citizens about what programs are available. [CP3] “To date, the use of genomic testing still seems too restricted in terms of territorial accessibility (…) there is great inequality of access across territories.” [CP4]
	Clinical practice	Fractured patient doctor relationship Organizational problems fracture the doctor-patient relationship (overload, time shortage, waiting lists, administrative tasks, infrastructure)/organizational problems due to resources shortage Lack of adequate communication with patients/citizens/communication failures with patient/citizens Low accurate information provided to patients/citizens Clinical organization Insufficient service delivery and coverage Lack of integration with primary care/lack of GPs involvement Lack of coordination between medical specialities Insufficient access to technology/diagnostics Dependence of access to high-cost technology Insufficient evidence to support implementation into clinical practice Tertiary prevention focus Lack of procedures and data standards Insufficient standardization of clinical and laboratory reporting for genomic tests Lack of harmonized clinical data quality standards Lack of interoperability of data and clinical data standards	“(…) creates great frustration both in the patient, who has to deal with a professional who merely enters data into a system, and in the doctor, who feels his profession has been betrayed. (…) this is a major obstacle to personalized medicine.” [HP3] “I think the big mistake that is being made (…) is the little involvement of primary care physicians.” [HP9] “The use of genetic testing (…) is not widespread in clinical practice. (…) even where there is evidence, it is probably not sufficient for the adoption of personalized prevention strategies (…).” [HP3] “Difficulties in the integration of data with existing health systems (…).” [HP5] “(…) information about the impact of not attending a screening is underestimated by the general population (…) communication about the risks of non-adherence is rather rhetorical. This is why it is necessary to work through health care providers. The issue of trust and the citizen/health worker relationship is a central issue.” [CP4]
Research	Scientific strategy	Insufficient research on prevention Insufficient research on prevention Lack of incentives for research teams working on prevention Insufficient research to demonstrate benefit and clinical utility Lack of robust evidence reduce credibility and uptake, create scepticism and raise concerns about efficacy, potential harms and cost-effectiveness among health providers, policymakers and general public Insufficient evidence to support implementation Lack of standards on prevention research Low awareness regarding quality standards	“The lack of robust evidence supporting personalized prevention strategies (…) that can demonstrate efficacy and safety, reduces the credibility of these interventions and can impact the uptake of these strategies. (…) create scepticism among healthcare providers, policymakers, and the general public.” [PM5] “(…) in the field of treatment there are gold standard in terms of methodology and good practice (…) in the field of prevention, nothing equivalent exists (…).” [R2]
	Scientific funding	Insufficient funding streams Lack of funding for prevention research Insufficient economic incentives lead to lack of motivation and/or interest in developing prevention research Low interest from industry Low investment in large cohorts Low investment in genomic research Lack of vision for a long-term sustainability of data generated by healthcare systems	“Among the main problems has always been the lack of large funding in the area of research for personalized prevention aimed at building large studies. (…) funding has always been concentrated in the area of therapy.” [R4] “(…) people are not thinking about the sustainability and the long-term monitoring of the data that are being generated by the healthcare system (…)” [R5]
Implementation	Translational gaps	Lack of regulatory frameworks Lack of a regulatory system for translation of prevention research outputs Scientific knowledge is not sufficiently translated into clinical guidelines Lack of organization and harmonization of standards for personalized prevention High dependence of international guidelines or consortium Length of time and costs of translation Personalized prevention strategies take time to enter into practice Personalized prevention strategies are perceived as expensive Complexicity of personalized prevention operationalization Low promotion of implementation due to misconstrued concept of personalized prevention as not ready for healthcare Complexity of personalized prevention implementation (many layers of intervention) Lack of an effective communication of scientific evidence to non-scientific audiences	“(…) very little activity in the field of HTA for assessing the power of risk scores when compared to other clinical outcomes.” [R5] “We still have a huge problem for lack of verification of the data sets that come from the healthcare domain (…) the lack of a clear regulatory route for medical devices.” [R5] “(…) there is a lot of knowledge but it is not in the guidelines. (…) we do nothing because it is not in the guidelines.” [R1] “(…) for biomarkers risk scores and AI solutions healthcare systems are still not aware of the importance of funding for validation of these tools to bring them to clinical practice or technical decision-making process.” “Genetics-based screenings are expensive and difficult to implement in a short time frame (…)” [HP9] “(…) personalized medicine is not perceived as the medicine of the moment but rather as an emerging research field with potential benefits in the future (…).” [HP6] “(…) an issue to overcome is still the complexity of personalized prevention. (…) you give someone a pill and the person can get better. Personalized prevention is linked to behaviors of healthy people, which are very difficult to control.” [PM2] “The low uptake of personalized strategies is certainly due to a lack of perceived benefit of these, but a real lack of evidence is also a cause. (…) the potential of personalized medicine has been much hyped, promising great benefits always just around the corner. This corner, however, has not yet been well materialized and defined.” [HP3]
	Synergies between healthcare, research and industry	Resistance to collaboration Inertia to collaborate with other sectors Absent incentives for collaboration with other sectors Resistance to collaborate with industry Gap between research and entrepreneurship	“(…) health sector is not proactive enough in cooperating with other sectors. (…) there is no time allocation or financial or human resources allocation nor mandate or responsibility to actively network and liaise with other sectors.” [PM2] “Evidence for personalized prevention has existed for many years (…), as well as recommendations (…). However, in practice, things do not happen due to the inertia of healthcare systems. [HP6] “(…) getting academics to understand entrepreneurship. [R3]
	Ethical, legal and societal issues (ELSI)	Lack of data governance and reporting regulation Lack of a legal and regulatory framework for health data Lack of governance, legal and ethical frameworks Lack of ELSI guidelines for reporting research findings to patients Data protection issues Data privacy and security issues Insecurity regarding data protection Guarantee compliance with GDPR Discretionary adoption of GDPR at the national level relative to access to genomic and health data	“(…) problems with the governance, legal and ethical there are many questions unanswered,” [PM3] “(…) report back to the participant, but there is no guideline on it.” [R1] “(…) people do not think the systems in place are robust enough from a cyber-security perspective (…).” [PM3] “GDPR has been adopted differently by different member countries, which is certainly an obstacle to the uniform development of research.” [R4]
Awareness, education and literacy	Policymakers	Lack of awareness and literacy Low awareness and literacy of policymakers regarding personalized prevention Low awareness of policymakers regarding personalized prevention benefits Perception of personalized prevention approaches still in the research realm Lower perception of the positive impact of personalized preventive strategies in health outcomes Low political interest Asynchrony between health outcomes and political cycles Political influence on the allocation of resources/political determinants of health	“I think that what is lacking is a greater perception on the part of political decision-makers, of the importance of prevention strategies, with regard to their results, the health gains that the population will have.” [PM4] “When we talk about prevention strategies, we are talking about strategies whose results we will only be able to measure in the medium-long term. And if we are talking about political cycles of 4–5 years (…) they are not very attractive for a politician to implement.” [PM4] “Evidence needs to be better communicated to policymakers; we are bombarded with too much information (…). (…) we still need to have the best scientific evidence to date and how we can act on it (…).” [PM4] “We need to shift the perception, particularly among politicians, policymakers (…) that personalized prevention, along with public health in general, is a burden on the healthcare system” [PM5] “It is very important to convey the concept that new technologies in the area of personalized prevention are not the preserve of a few from the scientific world, but are essential tools for everyone, this awareness can stimulate need and promote investment.” [R4]
	Health professionals	Low level of awareness and insufficient knowledge Insufficient awareness of benefits of personalized prevention approaches/low perception of benefits and risks Perception of personalized prevention approaches still in the research realm Perception of personalized prevention approaches as very costly Perception of personalized prevention approaches as an emergent research field Stigma of genetics as dangerous Low awareness of personalized prevention in public health professionals Insufficient training Insufficient training and information about personalized prevention approaches for clinical practice Insufficient knowledge of existing evidence Insufficient communication skills of health professionals Lack of training/knowledge about genetic information utility Lack of training of medical students in a prevention setting Lack of training on communication with patients General practitioners’ low health literacy level	“(…) health care providers are often the first ones who are unaware of the possibility of personalized prevention (…)” [CP3] “The perception of doctors and decision-makers in the healthcare system regarding personalized prevention as a field of research and something that is more of a future concept rather than something that can already be implemented.” [HP6] “(…) the lack of education is a major barrier. General practitioners are not fully aware of the advances that the medical world is making, due to a lack of training.” [HP3] “(…) the lack of specific training on doctor-patient communication strategies means that the doctor improvises when communicating with the patient/citizen.” [HP3]
	Citizens and patients	Low health literacy level and knowledge Low health literacy level of citizens Insufficient health information and literacy Lack of knowledge about the benefits and risks Lack of training/knowledge about genetic information utility Lack of knowledge hinders patients’ involvement Lack of a widespread transmission of knowledge beyond doctor-patient relation Unsatisfied patient information needs Lack of health literacy programs Lack of knowledge about current programs Lack of education for prevention Low level of awareness Lack of awareness of non-attendance impact Lack of awareness and/or knowledge about available personalized prevention strategies A perception shift is needed for general acceptance of personalized prevention Low awareness and/or understanding of health benefits of prevention Low recognition of personal relevance Misinformation/disinformation Misinformation Disinformation Information biases influence patient/citizens attitude	“(…) many individuals may not be aware of the availability, benefits, and importance of participating in screening programs or other personalized prevention initiatives (…) lack of trust and perceived benefits in healthcare systems, concerns about privacy, and perceived low personal relevance of personalized prevention strategies.” [PM5] “People only want to learn about a disease when they are directly or indirectly affected by it.” [CP2] “Communication about personalized medicine and the amount of information provided are insufficient [to citizens/patients] (…).” [HP6] “The information and advice source. (…) instead of asking qualified professionals for a second or third opinion, we [citizens/patients] ask a neighbor, a co-worker or, worse, the internet, without validating the source of the answer.” [CP1] “It depends on how we communicate with patients (…) Usually patients embrace the initiative in a good way, but it is crucial how the information comes through.” [HP9]
Personal attitudes	Health professionals	Resistance to change Resistance to change current medical practices Professional resistance Medical paternalism Lack of motivation/resistance to change established practice Professional conflicting interests Lack of trust on the promised benefits of personalized prevention	“I believe that there is resistance from various sectors to these [preventive] programs. There can be competition for the same resources, at the limit” [PM1] “(…) the paternalistic attitude of the medical profession and the limited involvement of patients, and especially citizens, in co-creating pro-health solutions.” [HP6]
	Citizens and patients	Stigma Fear of health data misuse Fear of discrimination Genetic data stigma-related Perceived stigma Fear and disconfort Fear of results Fears of health risks (radiation exposure) Invasive nature of screenings Patients discomfort Inconvenience for patients Citizens feels uncertainty Insecurity Lack of confidence Negative psychosocial impacts of results/information Religious beliefs Lack of motivation Lack of motivation for prevention due to focus on treatment attitude Lack of patients’ motivation Lack of trust Insufficient patient empowerment Incapacity of family members involvement	“(…) there is a stigma on the use of genetics that may lead the community to a non-accepting stage.” [HP8] “(…) lack of awareness of the great advantage of an early diagnosis (…) combined with fear of finding something in the screening. (…) And then the inconvenience of the screening itself (…)” [CP2] “The psychological repercussions of screening results on patients. How will genetic-based risk information be handled.” [HP5] “(…) For prevention, you are healthy, you say why should I bother? So, it is also a personal attitude of healthy citizens that is not helping implementation of prevention.” [R1] “(…) reduced trust in doctors, the healthcare system, and science (…) among a large portion of the population.” [HP6]

PM, Policymaker; HP, Health Professional; R, Researcher; CP, Citizen/Patient.

As anticipated, some barriers were relevant to more than one domain. Although all stakeholder groups contributed to the thematic analysis, some thematic categories were more prominently discussed by specific stakeholder groups according to their professional roles, expertise, or lived experiences within the implementation context of personalized preventive interventions. This likely reflects the different institutional roles, professional responsibilities, expertise, and lived experiences of informants regarding the implementation of personalized preventive interventions. The illustrative quotes presented in Table 1 were selected to exemplify themes and sub-themes and do not fully represent the overall richness or distribution of perspectives across the complete qualitative dataset.

#### Healthcare systems

The current healthcare strategy focused on disease treatment as opposed to prevention, was perceived by many stakeholders from every stakeholder group as a major constraint to a wider adoption of PPI. Stakeholders pointed out that the predominance of the curative model over prevention in clinical services impacts on multiple building blocks of healthcare systems, including financing, a critical factor for policy implementation. Stakeholders considered that current investment in personalized prevention and health promotion is insufficient, partly due to a strong competition with other priorities and demands for public investment in health. The inexistence of cost–benefit analysis for PPI, and models for health technology assessment (HTA), a prerequisite for the market introduction of health devices and technology, that are not appropriate for prevention, contributes to a generalized scepticism of PP utility and effectiveness.

The lack of a solid strategy for PP, with a fragmented, mainly bottom-up implementation, and involving many uncoordinated actors with unclear roles and responsibilities, was also identified by interviewees. This lack of strategy leads to inequities in access to PPI, due to economic and other social issues (e.g., out-of-pocket expenses, reimbursement gaps, etc.). This can leave many people with a lower socioeconomic status behind.

Stakeholders further highlighted barriers to PPI presented by clinical services organization, namely the shortage of resources, the overload of health professionals with clinical and administrative tasks, and logistic problems, which altogether contribute to diminishing the trust between patients and health professionals. PPI relies on a set of expensive technologies, and some health systems have difficulty guaranteeing widespread access to these, with an impact on clinical services delivery and coverage, and ultimately hindering citizens and patients to benefit from its use. Interviewees also referred that key personal health data collected in the context of health service delivery (e.g., consultations, diagnostics, imaging, etc.), which enable the production of evidence to support tailored PPI, tends to suffer from a lack of standardization that limits its quality and usefulness.

#### Research

A main barrier referred at this level was the focus of current strategy and investment on disease treatment discovery, as opposed to research and innovation on prevention. The low interest in prevention research is eventually due to the time length required for high quality studies, and the very high costs of studying large population cohorts over long periods of time. There is also insufficient support to research teams to produce critical evidence for PPI implementation, including assessing clinical utility, exploring feasibility from an organizational standpoint, understanding its clarity for the citizen or assessing the readiness of the health professionals. Overall, due to underfunding and low incentives, current streams of research aren’t sufficiently focused on prevention, and the core research strays from PP.

#### Implementation

The length of time and costs of the translation of scientific findings to clinical practice for PPI, together with a lack of adequate regulatory frameworks and guidelines supporting health professionals, were gaps mentioned by stakeholders as holding back PPI wider implementation. Some stakeholders perceived PP as a complex and costly approach for health systems, with limited application in current clinical practice or promising great benefits that have not yet been materialized. This perception can be linked to health professionals and policymakers’ low awareness regarding sufficiently robust evidence to support the adoption of PPI.

Although synergies between healthcare, research and industry were noted to be fundamental for implementation of PPI, they were also regarded as insufficient. Feelings of distrust can explain the perceived resistance from all sides, hindering the translation of scientific knowledge into clinical settings.

PPI raises ethical and legal issues that impact citizens and patients, especially when supported by genetic data. Stakeholders mentioned concerns in personal data sharing because of uncertainties regarding its ethical use, as well as a perceived lack of security and privacy of data in healthcare systems, which can be a major barrier to public acceptance, compromising its societal benefits.

#### Awareness, education, and literacy

A main barrier mentioned by almost all stakeholders was a generalized lack of awareness, education and literacy for personalized prevention, from health professionals, citizens and patients, and policymakers.

Low awareness of the possibilities of PPI from health professionals, particularly clinicians, is linked with a perception of PPI as still in the realm of research and not ready for implementation. Combined with a lack of education on PP concepts, and a need for training to better communicate with patients, it is an important hindering factor for its adoption in healthcare.

Citizens and patients also remarked the low health literacy level on PPI. Reduced capacity to judge the quality of information, specially found on the internet, has an impact on perceived relevance of PPI to prevent disease.

Policymakers, on the other hand, feel they lack sufficient evidence regarding the validity, usefulness and cost–benefit of PPI, particularly in comparison with other less costly strategies that have a demonstrated positive impact in the general health of the population.

#### Personal attitudes

For health professionals, a resistance to change current practices, together with competition for the same resources, contributes to the low uptake of PPI.

For citizens, lack of awareness and a low health literacy level, lead to little understanding of benefits of early diagnosis, fears due to incidental findings or stigmatization, reducing adherence to PPI. There is a strong lack of motivation for screening, especially when they perceive themselves as healthy individuals for whom disease prevention is not an urgent concern.

### Survey outcomes

#### Participants characteristics

The findings presented are drawn from 270 complete answers to the survey. Citizens and Patients represented the largest stakeholder group (36%), followed by Researchers (31%) and Health professionals (24%), with the lower number of Policymakers (9%) reflecting the difficulty to engage this group. Respondents were generally highly educated, with half having doctoral studies, and only 11% with an educational degree below a master. Most respondents were from Portugal (29%), Italy (9%) and Sweden (8%) (see Supplementary Table 2 for participant characterization).

##### Main barriers to the adoption of personalized prevention approaches according to different stakeholders

**Figure 1:**
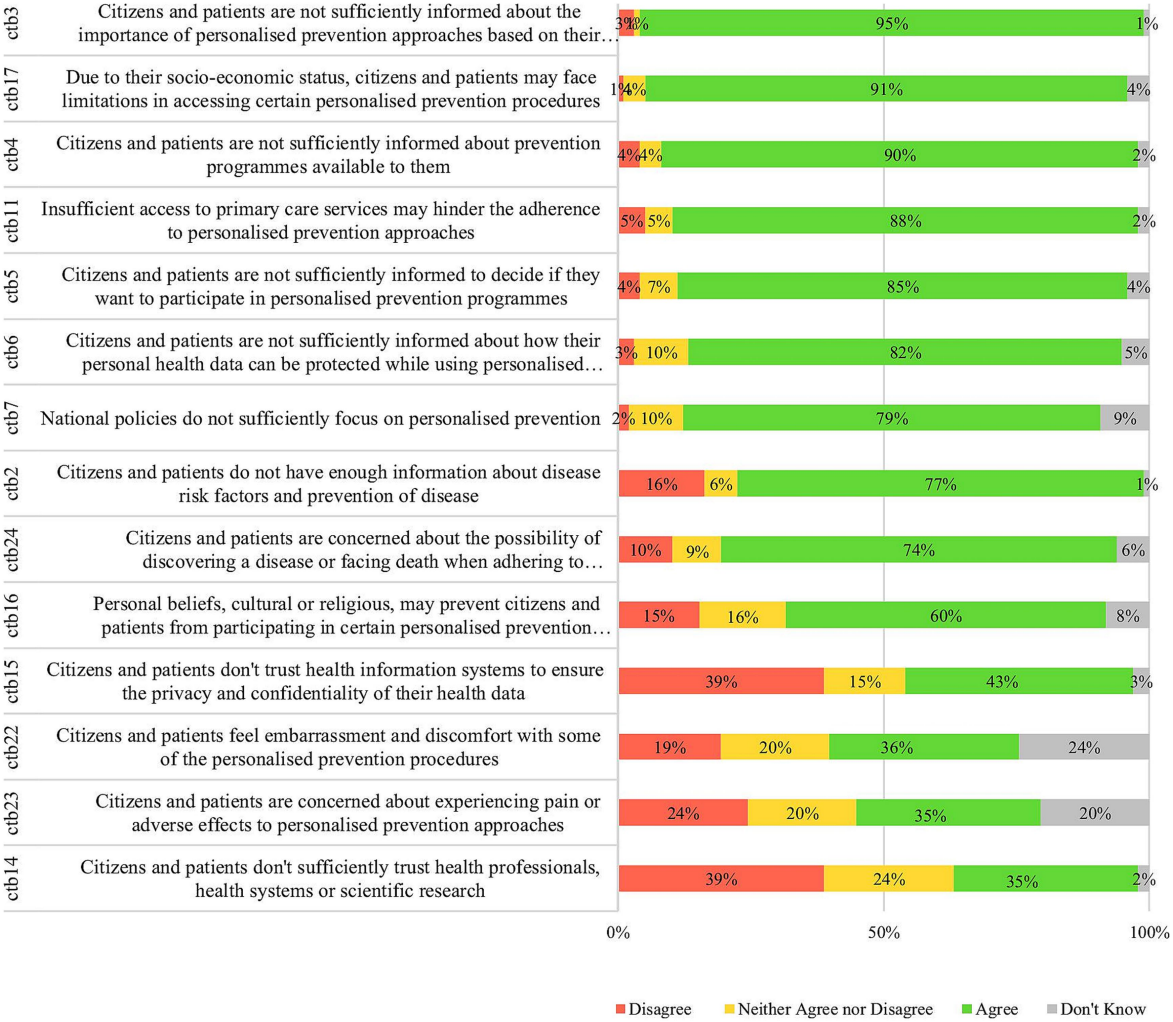
Perceived barriers and challenges to PPI, according to citizens/patients (Ctb).

**Figure 2:**
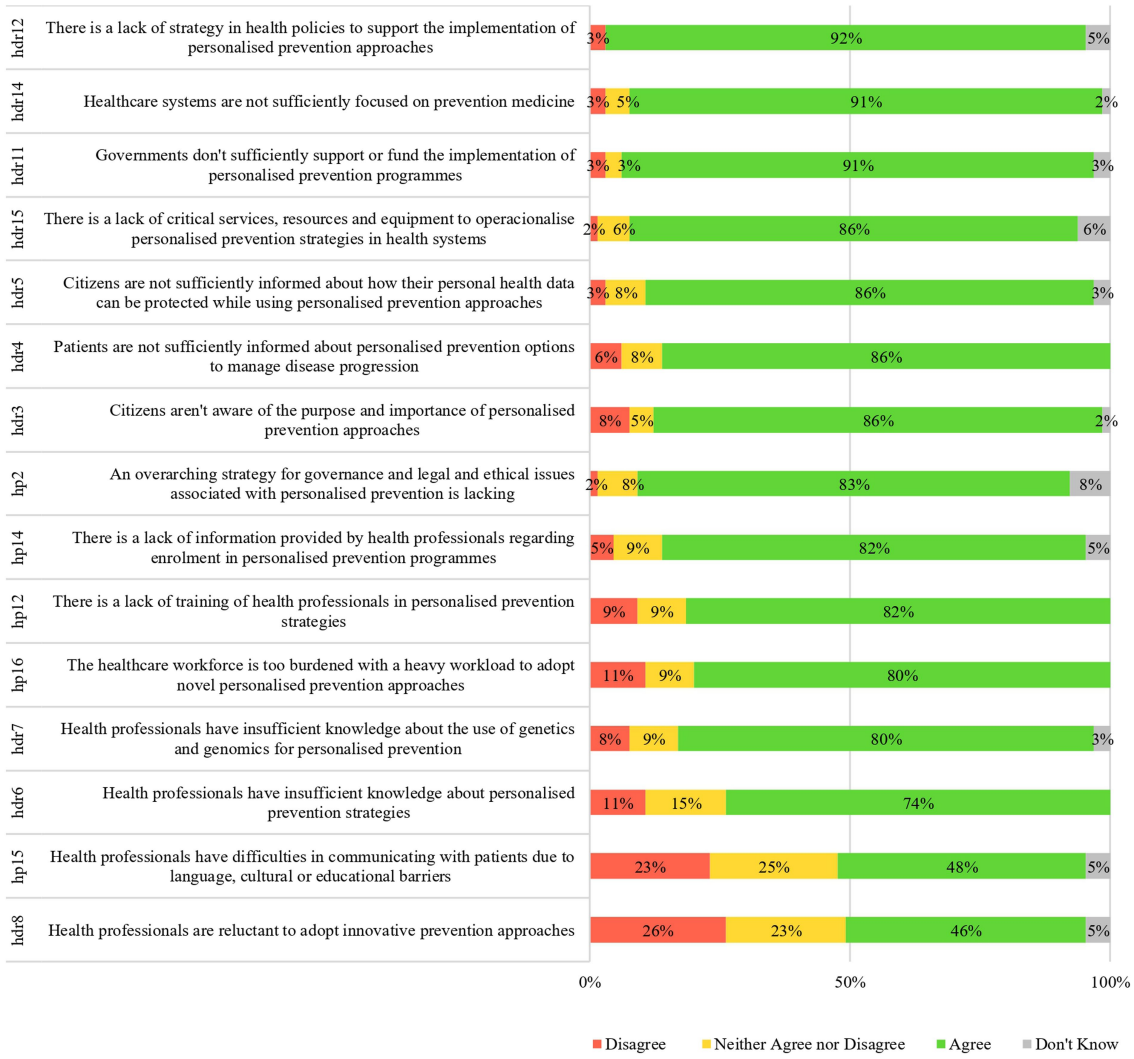
Perceived barriers and challenges to improve the health of citizens/patients based on PPI, according to health professionals (common items “hdr” and group-specific “hp”).

**Figure 3:**
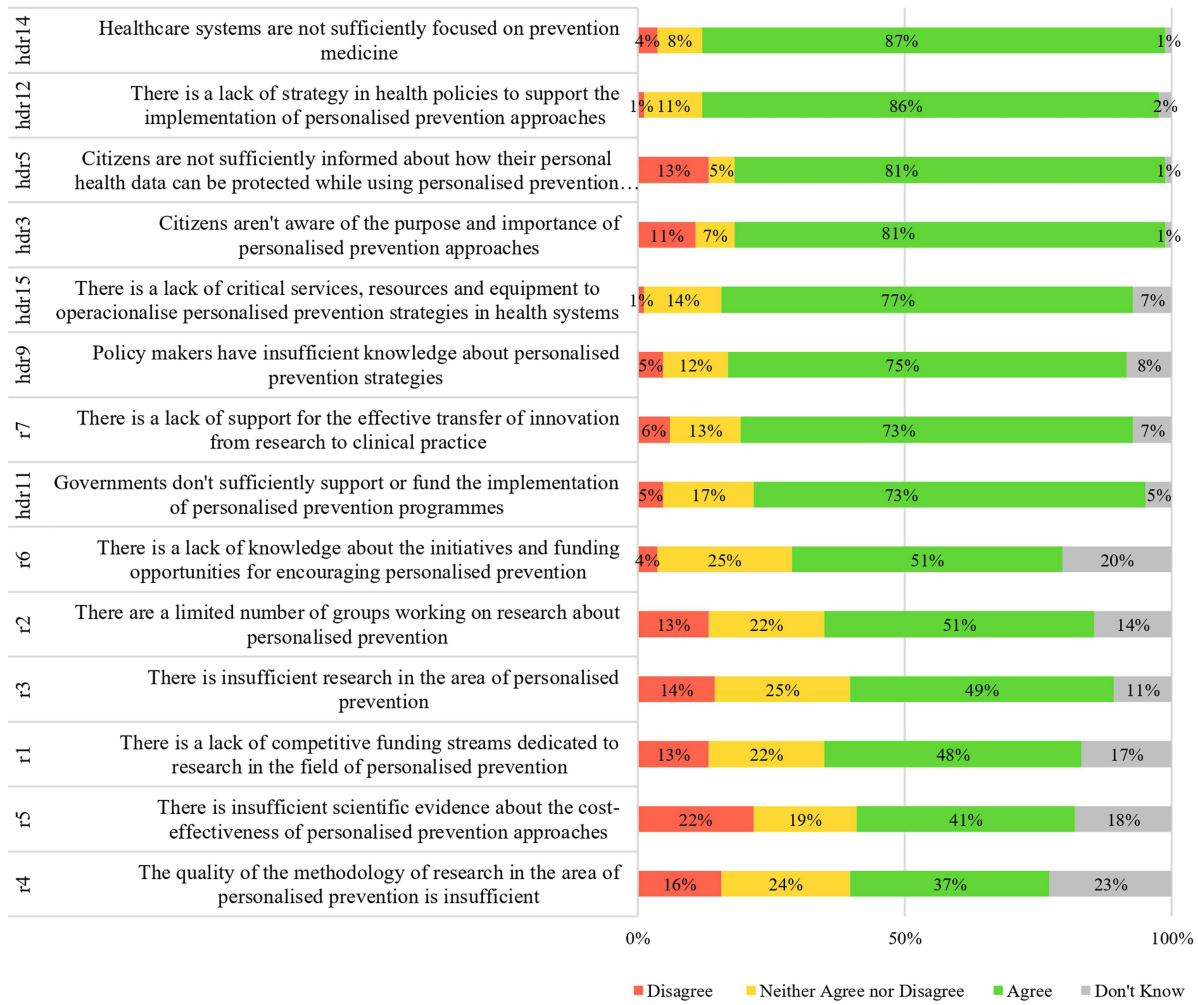
Perceived barriers and challenges to improve the health of citizens/patients based on PPI, according to researchers (common items “hdr” and group-specific “r”).

**Figure 4:**
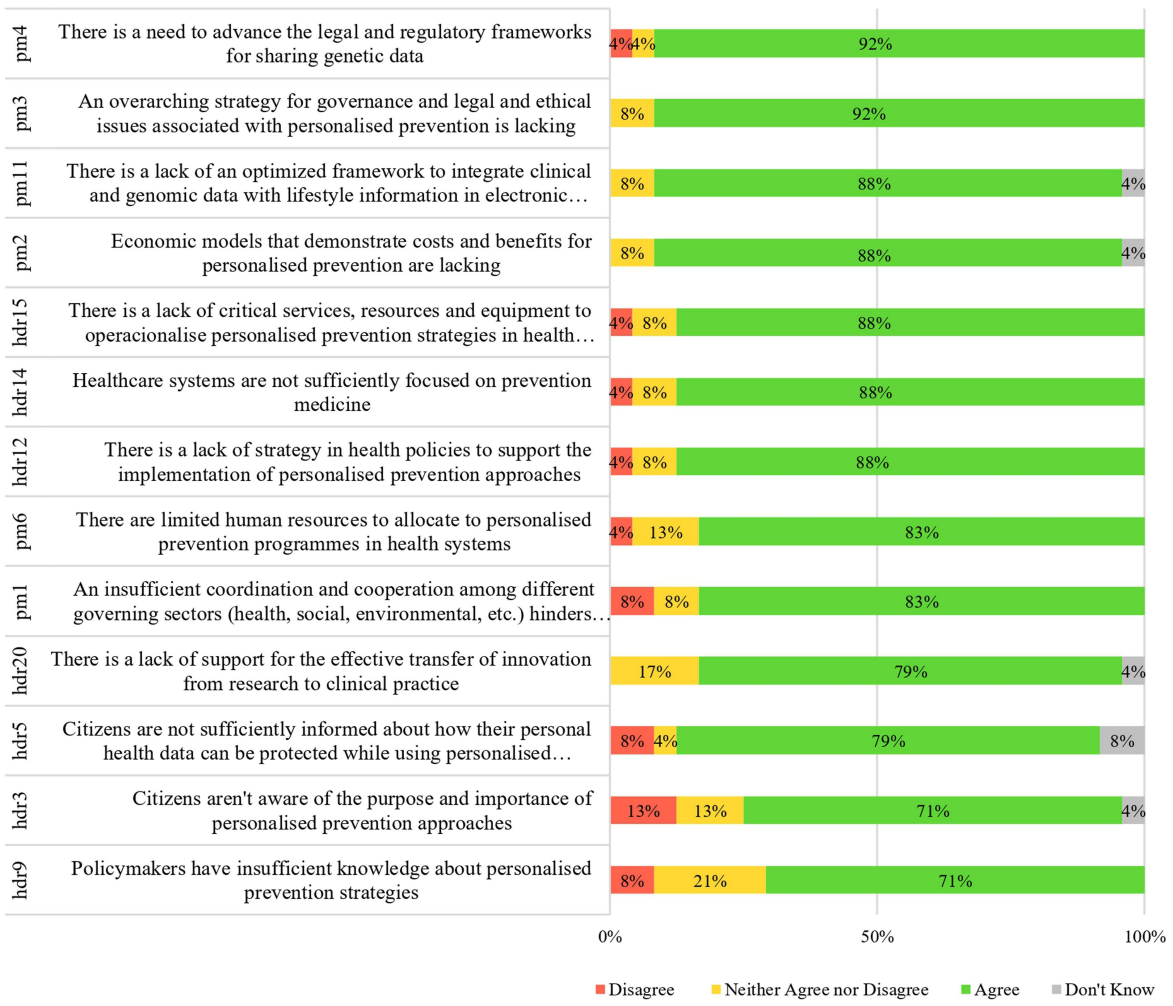
Perceived barriers and challenges to improve the health of citizens/patients based on PPI, according to policymakers (common items “hdr” and group-specific “pm”).

Barriers perceived by each stakeholder group were again categorized under the abovementioned five main levels described for expert interviews (Figures 1–4). Complete information is presented at Supplementary Tables 3–6.

#### Citizens and patients

For the 98 citizens and patients’ respondents, the most significant barriers to the adoption of PPI were related with *Awareness, education and literacy* and *Healthcare systems* (Figure 1). The large majority felt insufficiently informed about the importance of PP (ctb3), available prevention programs (ctb4), or generally to decide whether to enroll in PPI (ctb5).

For most, insufficient access to primary care services (ctb11), inequities in access due to socioeconomic status and financial limitations were perceived as significant barriers (ctb17), and a large proportion agreed that national policies do not focus sufficiently on personalized prevention (ctb7).

Personal feelings or fears faced by citizens and patients, such as embarrassment, discomfort or pain (ctb22 and ctb23), or fear of discrimination from providers (ctb21), were a concern for engaging in PPI in less than half of the participants, as were aspects related with lack of trust in health professionals, health systems, and scientific research (ctb14 and ctb15). However, over half of the respondents perceived personal beliefs or cultural/religious reasons as potential barriers (ctb16).

#### Health professionals

For the 65 health professionals surveyed (Figure 2), leading concerns at the *Healthcare systems* level were the lack of strategy and health policies (hdr12), as well as government support and funding (hdr11) for the implementation of PPI, and the low focus on preventive medicine (hdr14). The majority also highlighted insufficient services, resources and equipment (hdr1) as a critical barrier, and in particular a healthcare workforce too overloaded to adopt novel PPI (hp16).

At the *Awareness, education and literacy* level, most health professionals agreed that policymakers have insufficient knowledge about PP (hdr9), and that citizens and patients are unaware of the importance of PP (hdr3) and are not sufficiently informed about the available options to manage disease progression through PP (hdr4). A large majority of health professionals felt they lack training and knowledge on PPI (hp12 and hdr6) and lack knowledge about the use of genetics and genomics for PPI (hdr7). At the *Personal attitudes* level, less than half of health professionals agreed on being reluctant to adopt innovative PPI (hdr8) or felt they had challenges in communicating with patients due to language, cultural or educational barriers (hp15).

Major concerns at the *Implementation* level were the insufficient information for citizens about how their personal health data can be protected while using PPI (hdr5), and the lack of a framework to govern and address related legal and ethical issues (hp2).

#### Researchers

The majority of 83 researchers (Figure 3) agreed that *Healthcare systems* are not focused on prevention (hdr14), and that health policy strategies and adequate funding to support the implementation of PP are lacking (hdr12 and hdr11), with a scarcity of services, resources and equipment (hdr15).

At the *Awareness, education and literacy* level, researchers agreed that insufficient political knowledge (hdr9) is hindering support for the adoption of PPI. They also generally agreed with other stakeholders that there is a low awareness of citizens and patients regarding the purpose and importance of PPI (hdr3), as well as insufficient information about personal health data safeguards for personalized prevention use (hdr5). Most researchers highlighted the little support for transferring innovation from research to clinical practice (r7), while around half agreed that insufficient funding opportunities likely lead to few research groups and not enough research in personalized prevention (r6, r3, r1, r5).

#### Policymakers

The 25 Policymakers perceived main barriers related to *Implementation* and *Healthcare systems* (Figure 4). The vast majority recognized a lack of health system and public policies supporting PP (hdr12, hdr14), highlighting the need to advance legal and regulatory frameworks for sharing genetic data (pm4), as well a comprehensive strategy for governance and for legal and ethical issues associated with PP (pm3). Another pressing issue was the absence of economic models adequate for PPI (pm2), and the shortage of services and resources essential for the implementation of PPI (hdr15, pm6). Policymakers also emphasized the need for better integration of clinical and genetic data with lifestyle information in electronic health records (pm11). A large proportion recognized they lack sufficient knowledge about PPI (hdr9), and that there is a low level of awareness of citizens and patients about PPI and health data protection (hdr3, hdr5).

## Discussion

This study assessed and integrated the perspectives of four key stakeholder groups, from multiple European countries, on the barriers hindering the wide adoption of PPI in healthcare. The two-step multi-stakeholder consultation addressed a comprehensive range of structural, organizational, professional and other potential barriers.

The majority of experts, survey respondents, and members of the citizen and patient group reported higher education levels. This was likely influenced by the fact that the survey was conducted in English across all countries, reducing or excluding the participation of individuals with more limited English proficiency. Although this approach may have restricted the sociodemographic diversity of participants, we believe the complexity of the topic itself may also have contributed to the predominance of highly educated respondents. Addressing this limitation may require a substantial effort to improve public understanding of the PP concept, a need that was highlighted by this consultation.

The survey adopted a broad snowball dissemination strategy through *consortium*, professional, scientific, healthcare, and patient networks to maximize the diversity of perspectives across stakeholder groups. Participation relied on individual self-selection and no formal eligibility verification procedures were implemented beyond stakeholder self-identification and self-reported familiarity with the topic, potentially introducing a selection bias.

The aggregation of Likert-scale responses into broader agreement and disagreement categories may have reduced the granularity of participants’ perceptions and should therefore be interpreted as a descriptive simplification intended to facilitate the presentation of overall patterns. The questionnaire underwent internal review by members of the research team and consortium partners to assess clarity, relevance, and consistency with the study objectives, but formal validation procedures were not carried out potentially limiting the robustness and reproducibility of the instrument. This should be taken into consideration when interpreting the findings.

Interviewed stakeholders hailed from across Europe and worked for a range of European organizations. It should be noted that survey responses were primarily from the research team nationalities (Portugal, Italy and Sweden), reflecting the more successful dissemination of the survey in these countries. While some geographical bias cannot be ruled out, possibly limiting the full generalization of the findings to all European countries, the survey nevertheless gathered input from 26 countries, thus capturing a broad diversity of national healthcare, social and educational systems in qualitative terms. At the same time, healthcare systems and personalized prevention initiatives across Europe vary considerably regarding the organization of preventive services, governance structures, digital infrastructure, implementation pathways, healthcare financing models, and the maturity of PM and PP strategies (13, 16). Although these contextual differences were not specifically explored within the scope of this study, they may contribute to shaping how barriers to PPI implementation are perceived and prioritized across countries and stakeholder groups.

Importantly, the survey findings generally reinforced and complemented the barriers perceptions from interviewed informants across Europe, as well as concerns previously reported in the literature. Generally, there was overall consistency between the barriers identified in the interviews and the agreement levels reported by survey respondents across the four stakeholder groups. Some variations in the relative perceived importance of barriers across stakeholder groups were observed, likely reflecting their different institutional roles, professional responsibilities, expertise, and lived experiences regarding PPI implementation. Among citizens and patients, as well as policymakers, some barriers were perceived as particularly highlighted in light of their specific roles in the implementation of PPI as end-users and policymakers, respectively. This interpretation was informed by the overall patterns observed across stakeholder groups in the survey findings and by the complementary perspectives emerging from the qualitative data. At the same time, these stakeholder groups also acknowledged many of the challenges highlighted by healthcare professionals and researchers, although with different perceived levels of relevance and priority. Several barriers to a wider adoption of PPI were consistently highlighted across stakeholder groups and across both phases of the study particularly those related to *healthcare systems*, *implementation*, and *awareness, education, and literacy levels* identified through the thematic analysis of the interviews and subsequently reinforced by the survey findings.

At a macro level, a recurring concern across stakeholder groups was the perception that healthcare systems remain predominantly oriented toward treatment rather than prevention, which participants frequently associated with limited strategic and political support for preventive approaches. Both aspects are interlinked and have significant implications for the deployment of PPI, namely the required funding allocation, the provision of adequate resources (personnel, infrastructure, etc.), the establishment of reimbursement processes and guaranties of equitable access (6). Moreover, stakeholders also noted a lack of a governance framework to ensure coordination between all actors involved, and to fully address legal and ethical issues raised by PP, as factors hindering PPI adoption.

A key issue highlighted by stakeholders is equity of access to PPI, inadequately addressed within the current model of delivery. Citizens and patients highlighted concerns about personal costs of PPI (17), and the potential exclusion of disadvantaged groups, echoing previously recognized concerns of socioeconomic and geographical disparities disproportionately affecting individuals of lower socioeconomic status (18). Reimbursement and other related issues need to be carefully addressed to ensure equitable access in PPI implementation, to avoid the exacerbation of persistent health inequalities (17, 19).

Unresolved ethical and legal issues related to health data security and individual data protection provided by healthcare systems, particularly for genetic information, was a shared concern from policymakers, healthcare professionals and researchers. Citizens and patients were divided on whether they regarded health systems as trustworthy protectors of privacy and confidentiality of their data. To promote trust in the system, and to avoid public scepticism about data sharing and reuse, ensure transparency in the use of genetic and other sensitive health data is needed. Establishing governance frameworks, clarifying roles and responsibilities, and developing clear guidelines to standardize data collection, management and use, may contribute to fostering public acceptance of personalized prevention (16). Patients are generally interested in ensuring that sharing their data brings benefits at both the individual and societal levels (20). Their involvement as patient advocates in the governance of large-scale initiatives involving data sharing may foster greater patient participation by truly meeting their expectations and needs, as well as promoting trust within the general population (16, 21).

A lack of awareness, education and literacy about personalized prevention among citizens/patients, health professionals and policymakers was generally perceived, which hinders their capacity to adhere to PPI or support its implementation in healthcare systems. Healthcare professionals underlined insufficient awareness to effectively implement PPI, with few opportunities and time to acquire knowledge and novel competencies that are essential to integration of PPI in clinical practice (22). Previous studies have identified cross-disciplinary education and hands-on training in genetics, pharmacology, bioinformatics and other areas as important strategies to promote PPI implementation in clinical practice (3, 21–23). Furthermore, healthcare professionals need to be trained to communicate effectively complex PM concepts, as they are the first line of communication with patients, a reliable source of information, and important intermediaries to PPI enrolment (24). To address these educational gaps, there is a crucial need of cross-disciplinary competency frameworks, supported by comprehensive guidelines and standards, as well as accessible resources (21, 22, 25).

Limited knowledge and awareness of policymakers about the potential of PP was underlined as a major challenge to resolve. Policymakers generally perceived that robust scientific evidence for PPI effectiveness is still lagging, and that PPI clinical utility and cost-benefits is not demonstrated. As a result, they may prioritize short-term treatment outcomes over the long-term benefits of PP, in a policy arena highly competitive for limited resources, and within political cycles of 3–5 years. This suggests the importance of a more effective strategy to communicate scientific evidence supporting preventive interventions to policymakers as a means of overcoming policy barriers. Interviewed informants highlighted the lack of adequate economic models to assess and demonstrate cost-benefits for PPI, contributing to scepticism about its utility and effectiveness (26, 27). Concerted efforts to promote a continuous research-implementation feedback-loop and improve collaboration and communication between researchers, health professionals and policymakers are key for wider PPI adoption (21).

The broader transition toward PM, including a wider adoption of prevention-oriented strategies, may raise societal challenges related to understanding, trust, participation, and policymaking (28). Without adequate knowledge and confidence to manage their health, citizens and patients will struggle to understand and accept PPI, due to the complexity of the concept, combined with personal attitudes and fears (18). It is therefore important to provide individuals at least a basic understanding that allows them to make informed decisions on PPI reflecting their personal values (28, 29). The development of comprehensive communication programs that promote awareness-raising initiatives (including public awareness campaigns, conferences, workshops, etc.) engaging patients and the general population has the potential to foster a common understanding of the value of personalized prevention, address the concerns of various stakeholders, and simultaneously share new knowledge and innovations, enhancing its public acceptance (21, 25). The current development of ongoing national, regional and local initiatives dedicated to personalized medicine, genomic medicine or health data sharing may support the implementation of these expanded communication programs, reaching a larger audience of stakeholders (4, 11).

## Conclusion

This study provides a multi-stakeholder perspective on the barriers perceived to hinder the wider adoption of PPI in European healthcare systems.

Our findings suggest that the implementation of PPI is influenced by a set of interconnected challenges across healthcare system, governance, implementation, and awareness-related domains, highlighting the multidimensional nature of personalized prevention adoption.

By integrating the perspectives of citizens and patients, healthcare professionals, researchers, and policymakers, the study contributes to a more comprehensive understanding of the factors that may influence the adoption of PPI and identifies areas where greater awareness, coordination, and stakeholder engagement may support implementation efforts within European healthcare systems.

At the national level, emerging strategies for genomic medicine and personalized healthcare may provide opportunities to support the broader integration and adoption of personalized preventive approaches, reflecting an ongoing transition toward more personalized and prevention-oriented healthcare paradigms (30). National initiatives are further supported by European-wide programs promoting large-scale research projects, FAIR data access, ethical, legal and social governance frameworks, regulatory platforms with shared standards and guidelines, and generally facilitating the implementation of personalized medicine at the global level.

This *momentum* may provide opportunities to foster dialogue among stakeholders and to support the development of more comprehensive and sustainable solutions, as the successful adoption of PPI will likely require balancing the expectations, priorities, and concerns of multiple actors in its implementation across Europe.

## Data Availability

The original contributions presented in the study are included in the article/Supplementary material, further inquiries can be directed to the corresponding author.

## Footnotes

1
https://prophetproject.eu/
