# Supplementary material for: A multi-stakeholder perspective on barriers to the adoption of personalized prevention in European healthcare systems
Source: Front Public Health. 2026 Jun 24;14:1817705. doi: 10.3389/fpubh.2026.1817705 (PMC13341729; doi:10.3389/fpubh.2026.1817705)
Supplement: Supplementary file 1 [file Supplementary_file_1.docx]

**SUPPLEMENTAL MATERIAL**

**Supplemental Material 1 - Interview Guides**

**Interview guide 1 – Policymakers (PM)**

| Target group: Health Authorities, health administrators and public health organizations involved in policy and decision making |
| --- |
| Context: There is a substantial evidence base showing that many disease prevention interventions, delivered within the health system as well as in partnership with other sectors, are highly cost-effective. Despite this evidence, the level of investment in disease prevention strategies remains low in many countries. |
| Questions |
| Q1. What are the (three) main aspects that you consider need to be addressed to overcome the low level of investment in personalised prevention strategies? |
| Q2.1. In your opinion what are the (three) main barriers for the implementation of personalized preventive strategies?  Q2.2. Do you find that the low adoption of preventive strategies, at any level of prevention, may be due to a perceived or real lack of sufficient evidence? |
| Q3. Do you find that access, accessibility, and equity issues are critical challenges for the implementation personalized prevention strategies?  Q3.1. Do you consider that equity is sufficiently considered in the implementation of personalised prevention strategies? |
| Q4. Regarding public acceptance and enrollment in personalised prevention strategies, such as screening programmes, what are the main (three) barriers that you consider that need further attention and action?  Q4.1. What are your suggestions to tackle and overcome them? |
| Q5. Much of the costs of poor health fall outside health care systems (e.g. long-term sickness work absence or premature retirement). What are the main barriers to engage other sectors in personalised prevention strategies?  Q5.1 Would their involvement be beneficial? |
| Q6. Are there any other barriers, challenges or gaps to a sustainable implementation of disease prevention strategies that you want to refer? |

**Interview guide 2 – Health Professionals (HP)**

| Target group: Health Professionals |
| --- |
| Context: Health professionals are responsible for the implementation of preventive strategies in daily practice and can contribute to citizens' awareness and improve their health literacy. |
| Questions |
| Q1. What are the (three) main aspects that need to be addressed to overcome the low level of adoption of personalised prevention strategies?  Q1.1. Do you find that a low adoption of personalised prevention strategies, at any level of prevention, may be due to a perceived or real lack of sufficient evidence? |
| Q2. What are the (three) main barriers to personalised prevention screening programs that need to be addressed to improve public acceptance and enrollment? |
| Q3. What is your perception regarding the level of adoption of personalized prevention in (your) daily practice?  Q3.1. What are the biggest challenges you face, in your daily practice, for the adoption of personalized prevention strategies? |
| Q4. What are the (three) major concerns or challenges regarding the engagement of (your) patients in personalised prevention strategies, such as genetic-based screening programmes? |
| Q5. Do you consider that citizens and patients understand personalised prevention strategies as relevant health interventions for early detection and effective treatment of diseases?  Q5.1. What is your perception regarding how useful healthcare professionals consider personalised preventive approaches for early detection of disease and effective treatment? |
| Q6. Do you find that a low awareness and training on the concepts that underlie personalised prevention strategies hinders its adoption and use by health professionals? |
| Q7. Do you think that information needs are currently met for the public/patients, and effective communication strategies are in place, ensuring that individual non-participation in prevention programs is a fully informed choice? |
| Q8. Do you find that health professionals need further training on the effective communication of complex concepts of personalised prevention strategies, to improve patient/citizen compliance? |
| Q9. What is your perception about the level of awareness and understanding of patients regarding personalised prevention options for managing disease progression? |
| Q10. Are there any other barriers, challenges, or gaps to a sustainable implementation of disease prevention strategies that you want to refer? |

**Interview guide 3 – Citizens and Patients (CP)**

| Target group**:** Citizens and Patients |
| --- |
| Context: Patients and citizens can strongly benefit from preventive health interventions. Nevertheless, there are barriers and challenges that need to be overcome to have empowered and healthier citizens. Patients’ views and beliefs about barriers to screening programs participation, the adoption of healthier habits and other preventive strategies are relevant to design strategies that meet their needs. |
| Questions |
| Q1. What are the main (three) benefits of personalised prevention health interventions, such as screening programmes for cardiovascular disease, cancer, and others? |
| Q2. What are the (three) main barriers that hamper public acceptance and enrollment in personalised prevention strategies, such as screening programmes? |
| Q3. Do you find that health systems and policies sufficiently support personalised prevention strategies as a tool to prevent disease? |
| Q4. Do you believe that citizens/patients have the tools (knowledge, skills, confidence) to change individual habits (e.g., physical inactivity, weight, smoking, etc.) with a positive impact in your quality of life and/or longevity? |
| Q5. Do citizens/patients have sufficient knowledge about the processes, risks and benefits, and potential outcomes of personalised prevention strategies?  Q5.1. What do you think is needed to improve the awareness and public acceptance to screening enrollment? |
| Q6. Is the general population fully aware of the impact of a screening non-attendance on their health results? |
| Q7. What are the main concerns or fears felt by citizens/patients that may hinder their participation in screening programmes or other personalised preventive approaches? |
| Q8. Do you think that an increased health literacy at the population level is needed to improve the adoption of personalised prevention strategies?  Q8.1. What are your suggestions to improve it? |
| Q9. Are there any other barriers, challenges or gaps to a sustainable implementation of disease prevention strategies that you want to refer? |

**Interview guide 4 – Researchers (R)**

| Target group: Research & Innovation Organizations |
| --- |
| Context: Research is essential to address current challenges and to identify facilitators for implementation as well as to identify new paths for prevention. However, premature translation of innovations in prevention can do more harm than benefit. |
| Questions |
| Q1. What are the (three) main aspects that you consider need to be addressed to overcome the low level of investment in personalised prevention strategies? |
| Q2. What is the level of alignment of your organization’s mission with preventive personalized medicine? |
| Q3. What are the main barriers to collect high quality evidence on the efficacy, effectiveness and efficiency to support the adoption of personalized approaches in prevention? |
| Q4. Do research organizations have a role and responsibility in promoting the understanding of novel findings, to facilitate the adoption of personalised prevention strategies? |
| Q5. What are the main gaps contributing to the low adoption of personalised prevention strategies, at any level of prevention, that can be tackled by the R&D organizations? |
| Q6. How can research organizations further optimize the EU investment in personalised preventive tools (new technologies and devices)? |
| Q7. Is there is a lack of long-term data on effectiveness of many public health and health promoting interventions? If yes, how can this situation be overcome? |
| Q8. Are there any other barriers, challenges, or gaps to a sustainable implementation of disease prevention strategies that you want to refer? |

**Supplemental Material 2 – Survey**

**Methodological note:** For citizens and patients, the items were phrased in accessible language appropriate for this group (identified with the label “ctb”). For the other stakeholder groups, there was a set of common questions (labeled “hdr”) to all of the groups answered, and a set of questions specific for policymakers (labeled “pm”), health professionals (labeled “hp”) and researchers (labeled “r”). Labels are mentioned to assist item identification and are embedded in the question code.

[1] When answering this survey, do you consider yourself as:

Citizen and/or Patient *[ctb]*


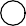

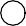

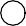

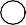


Health Professional (medical doctor, nurse, genetic counselor, other) *[hp]*

Researcher *[r]*

Health Administrator/Health Authority/Policy or Policymaker *[pm]*

[2] Do you suffer from any chronic disease*^^[[1]](#footnote-1)^^?

Yes^+^


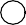

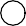


No

^+^Please specify_________________

**Section I**

[3] Have you ever heard of personalised prevention prior to this survey?

No^+^


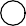

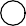

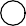

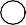


Yes, I have experienced a personalised prevention intervention before

Yes, I know someone who has experienced a personalised prevention intervention

Yes, because my work is related to healthcare (e.g., I am a healthcare professional, health administrator, policy maker, researcher, educator)


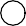
 Yes, I learned about this topic through other ways

^+^Please specify________

[4] Do you believe that personalised prevention is a beneficial health intervention to prevent disease, and reduce disability or mortality?

Yes,because^+^... No.


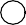

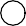


^+^Please select among the given options:


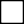
 It allows early diagnosis and more effective treatment.


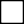
 It allows early detection of risk factors and adoption of preventive strategies.


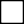
 It provides reassurance.


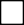
 Other^++^.

++ Please justify______________________

[5] Do you believe there are benefits in using pharmacogenomics for preventing adverse drug reactions and improving drug response?

Yes^+^


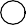

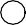

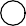


No^+^

Don't know

+Please justify_________________

**Section II**

***[Citizens/Patients group-specific items labeled “ctb”]***

**Section II:** In this section you are asked about your perceptions regarding the barriers and challenges to the use of personalised prevention.

[6] The following statements are related to barriers and challenges to the use of personalised prevention strategies to improve the health of citizens and patients. Please indicate your level of agreement with each sentence.

| Code | Item | Strongly Disagree | Disagree | Neither Agree  nor Disagree | Agree | Strongly Agree | Don’t Know |
| --- | --- | --- | --- | --- | --- | --- | --- |
| ctb1 | Citizens and patients don't have enough information to make informed decisions to maintain and improve their health | 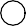 | 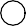 | 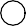 | 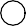 | 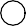 | 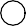 |
| ctb2 | Citizens and patients do not have enough information about disease risk factors and prevention of disease | 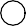 | 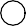 | 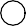 | 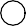 | 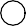 | 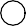 |
| ctb3 | Citizens and patients are not sufficiently informed about the importance of personalised prevention approaches based on their health indicators, biomarkers or their family health history | 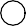 | 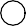 | 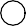 | 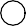 | 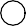 | 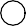 |
| ctb4 | Citizens and patients are not sufficiently informed about prevention programmes available to them | 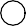 | 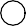 | 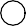 | 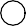 | 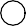 | 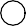 |
| ctb5 | Citizens and patients are not sufficiently informed to decide if they want to participate in personalised prevention programmes | 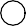 | 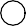 | 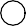 | 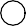 | 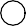 | 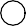 |
| ctb6 | Citizens and patients are not sufficiently informed about how their personal health data can be protected while using personalised prevention approaches | 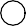 | 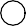 | 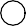 | 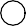 | 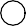 | 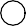 |
| ctb7 | National policies do not sufficiently focus on personalised prevention | 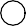 | 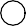 | 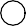 | 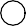 | 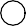 | 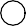 |
| ctb8 | Difficulties with reimbursement or insurance coverage may hinder the adherence of citizens and patients to personalised prevention programmes | 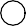 | 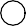 | 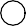 | 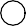 | 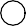 | 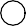 |
| ctb9 | Healthcare professionals do not sufficiently inform or recommend participation in personalised prevention approaches | 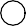 | 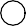 | 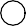 | 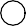 | 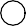 | 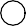 |
| cbt10 | Citizens and patients face significant financial difficulties to adhere to personalised prevention programmes because these have high costs | 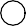 | 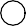 | 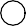 | 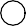 | 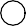 | 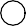 |
| ctb11 | Insufficient access to primary care services may hinder the adherence to personalized prevention approaches | 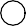 | 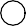 | 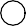 | 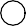 | 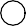 | 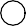 |

[7] The following statements are related to your feelings of trust and concerns regarding the use of personalised prevention strategies. Please indicate your level of agreement with each sentence.

| Code | Item | Strongly Disagree | Disagree | Neither Agree  nor Disagree | Agree | Strongly Agree | Don’t Know |
| --- | --- | --- | --- | --- | --- | --- | --- |
| ctb13 | Citizens and patients have difficulty in communicating with healthcare professionals due to language, cultural or educational barriers | 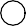 | 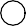 | 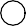 | 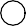 | 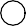 | 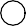 |
| ctb14 | Citizens and patients don't sufficiently trust health professionals, health systems or scientific research | 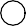 | 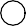 | 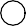 | 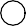 | 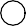 | 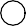 |
| ctb15 | Citizens and patients don't trust health information systems to ensure the privacy and confidentiality of their health data | 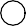 | 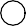 |  |  |  |  |
| ctb16 | Personal beliefs, cultural or religious, may prevent citizens and patients from participating in certain personalised prevention procedures |  |  |  |  |  |  |
| ctb17 | Due to their socio-economic status, citizens and patients may face limitations in accessing certain personalised prevention procedures |  |  |  |  |  |  |
| ctb18 | Citizens and patients have other pressing priorities, such as work-related responsibilities, family commitments, or other health concerns, which hinder their ability to prioritize adhesion to personalised prevention programmes |  |  |  |  |  |  |
| ctb19 | Citizens and patients have concerns regarding sharing their health data, including genetic data |  |  |  |  |  |  |
| ctb20 | Citizens and patients fear being stigmatized by their personal health information |  |  |  |  |  |  |
| ctb21 | Citizens and patients fear discrimination from providers of personalised prevention approaches |  |  |  |  |  |  |
| ctb22 | Citizens and patients feel embarrassment and discomfort with some of the personalised prevention procedures |  |  |  |  |  |  |
| ctb23 | Citizens and patients are concerned about experiencing pain or adverse effects to personalised prevention approaches |  |  |  |  |  |  |
| ctb24 | Citizens and patients are concerned about the possibility of discovering a disease or facing death when adhering to personalised prevention approaches |  |  |  |  |  |  |

***[Common items to Health Professionals, Researchers and Policymakers labeled “hdr”]***

**Section II:** In this section you are asked about your perceptions regarding the barriers and challenges to the use of personalised prevention

[8] The following statements are related to general barriers and challenges to the implementation of personalised prevention to improve the health of citizens and patients. Please indicate your level of agreement with each sentence.

| Code | Item | Strongly Disagree | Disagree | Neither Agree  nor Disagree | Agree | Strongly Agree | Don’t Know |
| --- | --- | --- | --- | --- | --- | --- | --- |
| hdr1 | Citizens have a low health literacy level |  |  |  |  |  |  |
| hdr2 | There is a lack of accurate information for citizens about disease risk factors and personalised prevention strategies |  |  |  |  |  |  |
| hdr3 | Citizens aren't aware of the purpose and importance of personalized prevention approaches |  |  |  |  |  |  |
| hdr4 | Patients are not sufficiently  informed about personalised prevention options to manage  disease progression |  |  |  |  |  |  |
| hdr5 | Citizens are not sufficiently  informed about how their  personal health data can be  protected while using  personalised prevention  approaches |  |  |  |  |  |  |
| hdr6 | Health professionals have  insufficient knowledge about personalised prevention strategies |  |  |  |  |  |  |
| hdr7 | Health professionals have  insufficient knowledge about the use of genetics and genomics for personalised prevention |  |  |  |  |  |  |
| hdr8 | Health professionals are  reluctant to adopt innovative prevention approaches |  |  |  |  |  |  |
| hdr9 | Policy makers have insufficient knowledge about personalised prevention strategies |  |  |  |  |  |  |
| hdr11 | Governments don't sufficiently support or fund the implementation of personalised prevention programmes |  |  |  |  |  |  |
| hdr12 | There is a lack of strategy in health policies to support the implementation of personalised prevention approaches |  |  |  |  |  |  |
| hdr13 | The traditional pharmaceutical company model does not encourage the implementation of personalised prevention  strategies |  |  |  |  |  |  |
| hdr14 | Healthcare systems are not  sufficiently focused on  prevention medicine |  |  |  |  |  |  |
| hdr15 | There is a lack of critical  services, resources and  equipment to operacionalise personalised prevention strategies in health systems |  |  |  |  |  |  |
| hdr16 | There is a lack of adequate  infrastructures for managing biological samples as well as health and related genomic data  to support personalised prevention programmes |  |  |  |  |  |  |
| hdr17 | There is insufficient research in the area of personalised prevention |  |  |  |  |  |  |
| hdr18 | There is insufficient scientific evidence about the cost-effectiveness of  personalised prevention  approaches* |  |  |  |  |  |  |
| hdr19 | There is a lack of knowledge about the initiatives and funding  opportunities for encouraging personalised prevention* |  |  |  |  |  |  |
| hdr20 | There is a lack of support for the effective transfer of innovation from research to clinical practice^*^ |  |  |  |  |  |  |

* Except to Researchers

***[Health Professionals group-specific items labelled “hp”]***

[9] The following statements are related to barriers and challenges to the implementation of personalised prevention in health systems. Please indicate your level of agreement with each sentence.

| Code | | Item | Strongly Disagree | Disagree | Neither Agree  nor Disagree | Agree | Strongly Agree | Don’t Know |
| --- | --- | --- | --- | --- | --- | --- | --- | --- |
| hp1 | An insufficient coordination and cooperation among different governing sectors (health, social, environmental, etc.) hinders the formulation of personalised prevention policies | |  |  |  |  |  |  |
| hp2 | An overarching strategy for governance and legal and ethical issues associated with personalised prevention is lacking | |  |  |  |  |  |  |
| hp3 | There is a need to advance the legal and regulatory frameworks for sharing genetic data | |  |  |  |  |  |  |
| hp4 | There are limited financial  resources available for  implementation of personalised prevention programmes in health systems | |  |  |  |  |  |  |
| hp5 | There are limited human  resources to allocate to  personalised prevention  programmes in health systems | |  |  |  |  |  |  |
| hp6 | There is a low level of  coordination between different levels of care, such as primary care and hospitals, hindering the  adoption of personalised  prevention strategies to avoid the onset, progression and recurrence of diseases | |  |  |  |  |  |  |
| hp7 | There is a low level of  coordination between public and private healthcare providers, hindering the adoption of  personalised prevention  strategies to avoid the onset, progression and recurrence of diseases | |  |  |  |  |  |  |
| hp8 | There is a lack of robust and secure Information and  Communications Technology (ICT) systems to ensure the accuracy, timely updating and accessibility to information relevant for personalised prevention (e.g., updated guidelines, personal health data, family health history, etc.) | |  |  |  |  |  |  |
| hp9 | There is a lack of optimized  frameworks to integrate clinical and genomic data with lifestyle information in electronic health records | |  |  |  |  |  |  |
| hp10 | Multidisciplinary teams,  including medical doctors,  nurses, psychologists, genetic counselors, and others, are not sufficiently involved in the implementation of personalised prevention strategies | |  |  |  |  |  |  |
| hp11 | Health professionals don't have a clear concept of personalised prevention | |  |  |  |  |  |  |
| hp12 | There is a lack of training of health professionals in  personalised prevention  strategies | |  |  |  |  |  |  |
| hp13 | There is a lack in training of health professionals on how to effectively communicate with patients about personalised prevention strategies | |  |  |  |  |  |  |
| hp14 | There is a lack of information provided by health professionals regarding enrolment in  personalised prevention  programmes | |  |  |  |  |  |  |
| hp15 | Health professionals have  difficulties in communicating with patients due to language, cultural or educational barriers | |  |  |  |  |  |  |
| hp16 | The healthcare workforce is too burdened with a heavy workload to adopt novel personalised prevention approaches | |  |  |  |  |  |  |
| hp17 | Citizens and patients face  significant financial difficulties to adhere to personalised prevention approaches that have high costs associated | |  |  |  |  |  |  |
| hp18 | There is insufficient health  reimbursement and/or insurance coverage for personalised programmes prevention | |  |  |  |  |  |  |
| hp19 | There is a low equity in access to personalised prevention programmes | |  |  |  |  |  |  |
| hp20 | Minorities have a low level of enrolment in personalised prevention programmes due to fear of stigma | |  |  |  |  |  |  |
| hp21 | Emotional barriers (e.g., fear, worry, anxiety) lead to low level of adherence to personalised prevention programmes | |  |  |  |  |  |  |

***[Policymakers group-specific items labelled “pm”]***

[10] The following statements are related to barriers and challenges to the implementation of personalised prevention in health systems. Please indicate your level of agreement with each sentence.

| Code | Item | | | Strongly Disagree | | Disagree | | Neither Agree  nor Disagree | | Agree | | Strongly Agree | | Don’t Know | |
| --- | --- | --- | --- | --- | --- | --- | --- | --- | --- | --- | --- | --- | --- | --- | --- |
| pm1 | | An insufficient coordination and cooperation among different governing sectors (health, social,  environmental, etc.) hinders the formulation of personalised prevention policies |  | |  | |  | |  | |  | |  | |  |
| pm2 | | Economic models that  demonstrate costs and benefits for personalised prevention are lacking |  | |  | |  | |  | |  | |  | |  |
| pm3 | | An overarching strategy for governance and legal and ethical issue associated with personalised prevention is lacking |  | |  | |  | |  | |  | |  | |  |
| pm4 | | There is a need to advance the legal and regulatory frameworks for sharing genetic data |  | |  | |  | |  | |  | |  | |  |
| pm5 | | There are limited financial  resources available for  implementation of personalised prevention programmes in health systems |  | |  | |  | |  | |  | |  | |  |
| pm6 | | There are limited human  resources to allocate to  personalised prevention  programmes in health systems |  | |  | |  | |  | |  | |  | |  |
| pm7 | | The healthcare workforce is too burdened with a heavy workload to adopt novel personalised prevention approaches |  | |  | |  | |  | |  | |  | |  |
| pm8 | | There is a low level of  coordination between different levels of care, such as primary care and hospitals, hindering the adoption of personalised  prevention strategies to avoid the onset, progression and recurrence of diseases |  | |  | |  | |  | |  | |  | |  |
| pm9 | | There is a low level of  coordination between public and private healthcare providers, hindering the adoption of  personalised prevention  strategies to avoid the onset, progression and recurrence of diseases |  | |  | |  | |  | |  | |  | |  |
| pm10 | | There is a lack of a robust and secure Information and Communications Technology (ICT) systems to ensure the accuracy, timely updating and accessibility to information relevant for personalised prevention (e.g. updated guidelines, personal health data, family health history, etc.) |  | |  | |  | |  | |  | |  | |  |
| pm11 | | There is a lack of an optimized frameworks to integrate clinical and genomic data with lifestyle  information in electronic health records |  | |  | |  | |  | |  | |  | |  |
| pm12 | | There is a lack of competitive funding streams dedicated to research in the field of personalised prevention |  | |  | |  | |  | |  | |  | |  |
| pm13 | | There are a limited number of groups working on research about personalised prevention |  | |  | |  | |  | |  | |  | |  |
| pm14 | | Multidisciplinary teams,  including medical doctors,  nurses, psychologists, genetic counselors, and others, are not sufficiently involved in the implementation of personalised prevention strategies |  | |  | |  | |  | |  | |  | |  |
| pm15 | | Citizens and patients face  significant financial difficulties to adhere to personalised prevention approaches that have high costs associated |  | |  | |  | |  | |  | |  | |  |
| pm16 | | There is insufficient health  reimbursement and/or insurance coverage for personalised programmes prevention |  | |  | |  | |  | |  | |  | |  |
| pm17 | | There is low equity in access to personalised prevention programmes |  | |  | |  | |  | |  | |  | |  |
| pm18 | | Minorities have a low level of enrolment in personalised prevention programmes due to fear of stigma |  | |  | |  | |  | |  | |  | |  |

***[Researchers group-specific items labelled “r”]***

[11] The following statements are related to barriers and challenges to the implementation of personalised prevention in health systems. Please indicate your level of agreement with each sentence.

| Code | | Item | Strongly Disagree | Disagree | Neither Agree  nor Disagree | Agree | Strongly Agree | Don’t Know |
| --- | --- | --- | --- | --- | --- | --- | --- | --- |
| r1 | There is a lack of competitive funding streams dedicated to research in the field of  personalised prevention | |  |  |  |  |  |  |
| r2 | There are a limited number of groups working on research about personalised prevention | |  |  |  |  |  |  |
| r3 | There is insufficient research in the area of personalised prevention | |  |  |  |  |  |  |
| r4 | The quality of the methodology of research in the area of personalised prevention is insufficient | |  |  |  |  |  |  |
| r5 | There is insufficient scientific evidence about the cost-effectiveness of  personalised prevention  approaches | |  |  |  |  |  |  |
| r6 | There is a lack of knowledge about the initiatives and funding  opportunities for encouraging personalised prevention | |  |  |  |  |  |  |
| r7 | There is a lack of support for the effective transfer of innovation from research to clinical practice | |  |  |  |  |  |  |

[12] Are there any other important barriers and challenges to a sustainable adoption of personalised prevention strategies that you would like to refer?

________________________________

**Section III: In this section you are asked about your sociodemographic information**

[13] Gender:

Male Female

Non-binary Other+

Do not want to answer

+Please specify_________________

[14] Age:

18 - 29 yrs

30 - 39 yrs

40 - 49 yrs

50 - 59 yrs

60 - 69 yrs

70 - 79 yrs

80+ yrs

[15] In which country do you live in?

|  | Austria |
| --- | --- |
|  | Belgium |
|  | Bulgaria |
|  | Croatia |
|  | Cyprus |
|  | Czech Republic |
|  | Denmark |
|  | Estonia |
|  | Finland |
|  | France |
|  | Germany |
|  | Greece |
|  | Hungary |
|  | Ireland |
|  | Italy |
|  | Latvia |
|  | Lithuania |
|  | Luxembourg |
|  | Malta |
|  | Netherlands |
|  | Poland |
|  | Portugal |
|  | Romania |
|  | Slovakia |
|  | Slovenia |
|  | Spain |
|  | Sweden |
|  | UK |
|  | Other^+^ |

^+^Please specify_______________

[16] What is the highest educational level that you have completed?

Primary education Secondaryeducation Bachelor's degree Master's degree Doctoral studies

Other^+^

^+^Please specify___________

**Supplemental Table 1 – Characterization of interviewed experts^*^**

| **Stakeholder group** | **Number of experts (N)** | **Type of organization (N)** | **Main areas of expertise (N)** | **Countries**  **(N)** |
| --- | --- | --- | --- | --- |
| **Policymakers (PM)** | 5 | International health organization (3)  Health institution (1)  European Union institution (1) | Public Health (5) | Belgium (4)  Portugal (1) |
| **Health professionals (HP)**  Medical doctor (MD)  Pharmacist (P)  Genetic Counselor (GC) | 11  9  1  1 | Hospital (9)  Scientific and Clinical Organisation (1)  Medical Center (1) | MD Cardiology (1)  MD Clinical Pharmacology (2)  MD General Practitioner (2)  MD Oncogenomic (2)  MD Pneumonology (1)  MD Psychiatry (1)  GC Rare diseases (1)  P Clinical Pharmacology (1) | Italy (2)  Portugal (7)  Romania (1)  Spain (1) |
| **Citizens/Patients (CP)** | 4 | Patient Representatives Associations | Cancer (2)  Not disease-specific (1)  Rare diseases (1) | Italy (2)  Portugal (2) |
| **Researchers (R)** | 6 | University (6) | Genomics (4)  Neurology (1)  Public Health (1) | France (1)  Italy (1)  Netherlands (1)  Spain (1)  UK (2) |
| Total = 26 | | | | |

^*^Note: Experts provided their individual opinions not representative of any country or organization.

**Supplemental Table 2 - Characterization of survey participants by stakeholder group**

| **Characteristic** | **Citizens & Patients** | **Health professionals** | **Researchers** | **Policymakers** | **Total**  (n/%) |
| --- | --- | --- | --- | --- | --- |
| N/% total | 98 (36%) | 65 (24%) | 83 (31%) | 24 (9%) | 270 (100%) |
| **Gender** | **Citizens & Patients**  (n/%) | **Health professionals**  (n/%) | **Researchers**  (n/%) | **Policymakers**  (n/%) | **Total**  (n/%) |
| Male | 23 (23%) | 29 (45%) | 23 (28%) | 12 (50%) | 87 (32%) |
| Female | 75 (77%) | 36 (55%) | 58 (70%) | 12 (50%) | 181 (67%) |
| Non-binary | 0 (0%) | 0 (0%) | 0 (0%) | 0 (0%) | 0 (0%) |
| Other | 0 (0%) | 0 (0%) | 0 (0%) | 0 (0%) | 0 (0%) |
| Do not want to answer | 0 (0%) | 0 (0%) | 2 (2%) | 0 (0%) | 2 (1%) |
| N/% | 98 (100%) | 65 (100%) | 83 (100%) | 24 (100%) | 270 (100%) |
| **Age group** | **Citizens & Patients**  (n/%) | **Health professionals**  (n/%) | **Researchers**  (n/%) | **Policymakers**  (n/%) | **Total**  (n/%) |
| 18 - 29 y | 8 (8%) | 5 (8%) | 11 (13%) | 0 (0%) | 24 (9%) |
| 30 - 39 y | 17 (17%) | 15 (23%) | 18 (22%) | 4 (17%) | 54 (20%) |
| 40 - 49 y | 28 (29%) | 15 (23%) | 20 (24%) | 7 (29%) | 70 (26%) |
| 50 - 59 y | 30 (31%) | 17 (26%) | 16 (19%) | 10 (42%) | 73 (27%) |
| 60 - 69 y | 11 (11%) | 13 (20%) | 13 (16%) | 3 (13%) | 40 (15%) |
| 70 - 79 y | 4 (4%) | 0 (0%) | 4 (5%) | 0 (0%) | 8 (3%) |
| 80+ y | 0 (0%) | 0 (0%) | 1 (1%) | 0 (0%) | 1 (<1%) |
| N/% | 98 (100%) | 65 (100%) | 83 (100%) | 24 (100%) | 270 (100%) |
| **Educational level** | **Citizens & Patients**  (n/%) | **Health professionals**  (n/%) | **Researchers**  (n/%) | **Policymakers**  (n/%) | **Total**  (n/%) |
| Primary education | 0 (0%) | 0 (0%) | 0 (0%) | 0 (0%) | 0 (0%) |
| Secondary education | 5 (5%) | 0 (0%) | 0 (0%) | 1 (4%) | 6 (2%) |
| Bachelor's degree | 14 (14%) | 5 (8%) | 3 (4%) | 1 (4%) | 23 (9%) |
| Master's degree | 57 (58%) | 26 (40%) | 13 (16%) | 9 (38%) | 105 (39%) |
| Doctoral studies | 21 (21%) | 34 (52%) | 67 (81%) | 13 (54%) | 135 (50%) |
| Other | 1 (1%) | 0 (0%) | 0 (0%) | 0 (0%) | 1 (<1%) |
| N/% | 98 (100%) | 65 (100%) | 83 (100%) | 24 (100%) | 270 (100%) |
| **Country** | **Citizens & Patients**  (n/%) | **Health professionals**  (n/%) | **Researchers**  (n/%) | **Policymakers**  (n/%) | **Total**  (n/%) |
| Austria | 4 (4%) | 0 (0%) | 4 (5%) | 0 (0%) | 8 (3%) |
| Belgium | 5 (5%) | 1 (2%) | 7 (8%) | 0 (0%) | 13 (5%) |
| Bulgaria | 7 (7%) | 5 (8%) | 3 (4%) | 1 (4%) | 16 (6%) |
| Croatia | 0 (0%) | 0 (0%) | 0 (0%) | 0 (0%) | 0 (0%) |
| Cyprus | 1 (1%) | 0 (0%) | 0 (0%) | 0 (0%) | 1 (<1%) |
| Czech Republic | 0 (0%) | 0 (0%) | 1 (1%) | 1 (4%) | 2 (1%) |
| Denmark | 0 (0%) | 0 (0%) | 1 (1%) | 1 (4%) | 2 (1%) |
| Estonia | 1 (1%) | 3 (5%) | 4 (5%) | 0 (0%) | 8 (3%) |
| Finland | 1 (1%) | 1 (2%) | 5 (6%) | 1 (4%) | 8 (3%) |
| France | 6 (6%) | 1 (2%) | 2 (2%) | 0 (0%) | 9 (3%) |
| Germany | 2 (2%) | 0 (0%) | 2 (2%) | 0 (0%) | 4 (1%) |
| Greece | 0 (0%) | 8 (12%) | 1 (1%) | 0 (0%) | 9 (3%) |
| Hungary | 0 (0%) | 0 (0%) | 2 (2%) | 1 (4%) | 3 (1%) |
| Ireland | 2 (2%) | 0 (0%) | 0 (0%) | 0 (0%) | 2 (1%) |
| Italy | 11 (11%) | 6 (9%) | 5 (6%) | 1 (4%) | 23 (9%) |
| Latvia | 0 (0%) | 0 (0%) | 0 (0%) | 0 (0%) | 0 (0%) |
| Lithuania | 0 (0%) | 0 (0%) | 0 (0%) | 0 (0%) | 0 (0%) |
| Luxembourg | 0 (0%) | 0 (0%) | 1 (1%) | 0 (0%) | 1 (<1%) |
| Malta | 1 (1%) | 0 (0%) | 0 (0%) | 0 (0%) | 1 (<1%) |
| Netherlands | 3 (3%) | 4 (6%) | 9 (11%) | 1 (4%) | 17 (6%) |
| Poland | 0 (0%) | 0 (0%) | 0 (0%) | 1 (4%) | 1 (0,4%) |
| Portugal | 37 (38%) | 25 (38%) | 13 (16%) | 3 (13%) | 78 (29%) |
| Romania | 0 (0%) | 0 (0%) | 1 (1%) | 0 (0%) | 1 (<1%) |
| Slovakia | 1 (1%) | 0 (0%) | 0 (0%) | 0 (0%) | 1 (<1%) |
| Slovenia | 1 (1%) | 1 (2%) | 1 (1%) | 0 (0%) | 3 (1%) |
| Spain | 1 (1%) | 1 (2%) | 3 (4%) | 1 (4%) | 6 (2%) |
| Sweden | 3 (3%) | 6 (9%) | 6 (7%) | 6 (25%) | 21 (8%) |
| United Kingdom | 4 (4%) | 1 (2%) | 5 (6%) | 4 (17%) | 14 (5%) |
| Other countries (non-EU) | 7 (7%) | 2 (3%) | 7 (8%) | 2 (8%) | 18 (7%) |
| N/% | 98 (100%) | 65 (100%) | 83 (100%) | 24 (100%) | 270 (100%) |

**Supplemental Table 3 - Perceived barriers and challenges to personalised prevention interventions, according to citizens/patients** (group-specific items labelled “ctb”)

| Items | ctb1 | ctb2 | ctb3 | ctb4 | ctb5 | ctb6 | ctb7 | ctb8 | ctb9 | ctb10 | ctb11 | ctb13 | ctb14 | ctb15 | ctb16 | ctb17 | ctb18 | ctb19 | ctb20 | ctb21 | ctb22 | ctb23 | ctb24 |
| --- | --- | --- | --- | --- | --- | --- | --- | --- | --- | --- | --- | --- | --- | --- | --- | --- | --- | --- | --- | --- | --- | --- | --- |
| Strongly Disagree | 2% | 2% | 1% | 0% | 0% | 1% | 1% | 0% | 0% | 2% | 0% | 4% | 4% | 6% | 2% | 0% | 0% | 2% | 3% | 3% | 3% | 3% | 1% |
| Disagree | 16% | 14% | 2% | 4% | 4% | 2% | 1% | 4% | 2% | 4% | 5% | 14% | 35% | 33% | 13% | 1% | 9% | 13% | 13% | 21% | 16% | 21% | 9% |
| Neither Agree nor Disagree | 13% | 6% | 1% | 4% | 7% | 10% | 10% | 10% | 14% | 12% | 5% | 15% | 24% | 15% | 16% | 4% | 13% | 21% | 28% | 22% | 20% | 20% | 9% |
| Agree | 45% | 55% | 41% | 50% | 43% | 37% | 28% | 38% | 42% | 30% | 42% | 38% | 23% | 28% | 41% | 52% | 36% | 41% | 33% | 35% | 31% | 27% | 57% |
| Strongly Agree | 23% | 21% | 54% | 40% | 42% | 45% | 51% | 39% | 34% | 27% | 46% | 21% | 11% | 15% | 19% | 39% | 41% | 17% | 17% | 9% | 5% | 8% | 17% |
| Don't Know | 0% | 1% | 1% | 2% | 4% | 5% | 9% | 9% | 8% | 26% | 2% | 7% | 2% | 3% | 8% | 4% | 1% | 5% | 6% | 9% | 24% | 20% | 6% |
| Total | 100% | 100% | 100% | 100% | 100% | 100% | 100% | 100% | 100% | 100% | 100% | 100% | 100% | 100% | 100% | 100% | 100% | 100% | 100% | 100% | 100% | 100% | 100% |

**Supplemental Table 4 - Perceived barriers and challenges to personalised prevention interventions, according to healthcare professionals** (common items to health professionals, policymakers and researchers labelled “hdr”)

| Items | hdr1 | hdr2 | hdr3 | hdr4 | hdr5 | hdr6 | hdr7 | hdr8 | hdr9 | hdr11 | hdr12 | hdr13 | hdr14 | hdr15 | hdr16 | hdr17 | hdr18 | hdr19 | hdr20 |
| --- | --- | --- | --- | --- | --- | --- | --- | --- | --- | --- | --- | --- | --- | --- | --- | --- | --- | --- | --- |
| Disagree | 8% | 8% | 8% | 6% | 3% | 11% | 8% | 26% | 3% | 3% | 3% | 15% | 3% | 2% | 12% | 17% | 29% | 8% | 3% |
| Neither Agree nor Disagree | 14% | 9% | 5% | 8% | 8% | 15% | 9% | 23% | 9% | 3% | 0% | 20% | 5% | 6% | 12% | 20% | 15% | 17% | 6% |
| Agree | 75% | 82% | 86% | 86% | 86% | 74% | 80% | 46% | 85% | 91% | 92% | 57% | 91% | 86% | 74% | 57% | 48% | 62% | 83% |
| Don't Know | 3% | 2% | 2% | 0% | 3% | 0% | 3% | 5% | 3% | 3% | 5% | 8% | 2% | 6% | 2% | 6% | 8% | 14% | 8% |
| Total | 100% | 100% | 100% | 100% | 100% | 100% | 100% | 100% | 100% | 100% | 100% | 100% | 100% | 100% | 100% | 100% | 100% | 100% | 100% |

**Supplemental Table 4 (cont.) - Perceived barriers and challenges to personalised prevention interventions, according to healthcare professionals** (group-specific items labelled “hp”)

| Items | hp1 | hp2 | hp3 | hp4 | hp5 | hp6 | hp7 | hp8 | hp9 | hp10 | hp11 | hp12 | hp13 | hp14 | hp15 | hp16 | hp17 | hp18 | hp19 | hp20 | hp21 |
| --- | --- | --- | --- | --- | --- | --- | --- | --- | --- | --- | --- | --- | --- | --- | --- | --- | --- | --- | --- | --- | --- |
| Disagree | 0% | 2% | 8% | 5% | 12% | 5% | 3% | 6% | 3% | 5% | 14% | 9% | 6% | 5% | 23% | 11% | 11% | 8% | 11% | 12% | 14% |
| Neither Agree nor Disagree | 8% | 8% | 11% | 8% | 6% | 5% | 9% | 11% | 8% | 11% | 18% | 9% | 14% | 9% | 25% | 9% | 12% | 6% | 9% | 15% | 25% |
| Agree | 85% | 83% | 72% | 83% | 77% | 85% | 82% | 72% | 86% | 80% | 68% | 82% | 78% | 82% | 48% | 80% | 69% | 66% | 68% | 45% | 46% |
| Don't Know | 8% | 8% | 9% | 5% | 5% | 6% | 6% | 11% | 3% | 5% | 0% | 0% | 2% | 5% | 5% | 0% | 8% | 20% | 12% | 28% | 15% |
| Total | 100% | 100% | 100% | 100% | 100% | 100% | 100% | 100% | 100% | 100% | 100% | 100% | 100% | 100% | 100% | 100% | 100% | 100% | 100% | 100% | 100% |

**Supplemental Table 5 - Perceived barriers and challenges to personalised prevention interventions, according to researchers** (common items to health professionals, policymakers and researchers labelled “hdr” and group-specific items labelled “r”)

| Items | hdr1 | hdr2 | hdr3 | hdr4 | hdr5 | hdr6 | hdr7 | hdr8 | hdr9 | hdr11 | hdr12 | hdr13 | hdr14 | hdr15 | hdr16 | r1 | r2 | r3 | r4 | r5 | r6 | r7 |
| --- | --- | --- | --- | --- | --- | --- | --- | --- | --- | --- | --- | --- | --- | --- | --- | --- | --- | --- | --- | --- | --- | --- |
| Disagree | 13% | 12% | 11% | 5% | 13% | 2% | 2% | 25% | 5% | 5% | 1% | 5% | 4% | 1% | 7% | 13% | 13% | 14% | 16% | 22% | 4% | 6% |
| Neither Agree nor Disagree | 25% | 16% | 7% | 14% | 5% | 24% | 22% | 33% | 12% | 17% | 11% | 18% | 8% | 14% | 14% | 22% | 22% | 25% | 24% | 19% | 25% | 13% |
| Agree | 58% | 70% | 81% | 72% | 81% | 63% | 66% | 30% | 75% | 73% | 86% | 66% | 87% | 77% | 66% | 48% | 51% | 49% | 37% | 41% | 51% | 73% |
| Don't Know | 4% | 2% | 1% | 8% | 1% | 11% | 10% | 12% | 8% | 5% | 2% | 11% | 1% | 7% | 12% | 17% | 14% | 11% | 23% | 18% | 20% | 7% |
| Total | 100% | 100% | 100% | 100% | 100% | 100% | 100% | 100% | 100% | 100% | 100% | 100% | 100% | 100% | 100% | 100% | 100% | 100% | 100% | 100% | 100% | 100% |

**Supplemental Table 6 - Perceived barriers and challenges to personalised prevention interventions, according to policymakers** (common items to health professionals, policymakers and researchers labelled “hdr”)

| Items | hdr1 | hdr2 | hdr3 | hdr4 | hdr5 | hdr6 | hdr7 | hdr8 | hdr9 | hdr11 | hdr12 | hdr13 | hdr14 | hdr15 | hdr16 | hdr17 | hdr18 | hdr19 | hdr20 |
| --- | --- | --- | --- | --- | --- | --- | --- | --- | --- | --- | --- | --- | --- | --- | --- | --- | --- | --- | --- |
| Disagree | 17% | 21% | 13% | 4% | 8% | 17% | 17% | 17% | 8% | 4% | 4% | 13% | 4% | 4% | 8% | 8% | 21% | 8% | 0% |
| Neither Agree nor Disagree | 46% | 17% | 13% | 25% | 4% | 29% | 25% | 42% | 21% | 17% | 8% | 25% | 8% | 8% | 13% | 25% | 4% | 21% | 17% |
| Agree | 33% | 58% | 71% | 58% | 79% | 50% | 50% | 29% | 71% | 71% | 88% | 58% | 88% | 88% | 71% | 58% | 71% | 54% | 79% |
| Don't Know | 4% | 4% | 4% | 13% | 8% | 4% | 8% | 13% | 0% | 8% | 0% | 4% | 0% | 0% | 8% | 8% | 4% | 17% | 4% |
| Total | 100% | 100% | 100% | 100% | 100% | 100% | 100% | 100% | 100% | 100% | 100% | 100% | 100% | 100% | 100% | 100% | 100% | 100% | 100% |

**Supplemental Table 6 (cont.) - Perceived barriers and challenges to personalised prevention interventions, according to policymakers** (group-specific items labelled “pm”)

| Items | pm1 | pm2 | pm3 | pm4 | pm5 | pm6 | pm7 | pm8 | pm9 | pm10 | pm11 | pm12 | pm13 | pm14 | pm15 | pm16 | pm17 | pm18 |
| --- | --- | --- | --- | --- | --- | --- | --- | --- | --- | --- | --- | --- | --- | --- | --- | --- | --- | --- |
| Disagree | 8% | 0% | 0% | 4% | 0% | 4% | 4% | 4% | 4% | 4% | 0% | 4% | 13% | 4% | 8% | 8% | 8% | 13% |
| Neither Agree nor Disagree | 8% | 8% | 8% | 4% | 21% | 13% | 17% | 4% | 21% | 13% | 8% | 33% | 25% | 17% | 33% | 13% | 21% | 29% |
| Agree | 83% | 88% | 92% | 92% | 71% | 83% | 71% | 67% | 71% | 75% | 88% | 42% | 38% | 63% | 38% | 63% | 46% | 25% |
| Don't Know | 0% | 4% | 0% | 0% | 8% | 0% | 8% | 25% | 4% | 8% | 4% | 21% | 25% | 17% | 21% | 17% | 25% | 33% |
| Total | 100% | 100% | 100% | 100% | 100% | 100% | 100% | 100% | 100% | 100% | 100% | 100% | 100% | 100% | 100% | 100% | 100% | 100% |

1. *A chronic disease is a health condition that lasts 1 year or more and that require ongoing medical attention or limit activities of daily living, or both; for instance, cancer, diabetes, hypertension, stroke, heart disease, respiratory diseases, arthritis, obesity, and oral diseases.[1][2]

   References:

   [1]Centers for Disease Control and Prevention. About chronic diseases. https://www.cdc.gov/chronicdisease/about/index.htm. Accessed June 6, 2023.

   [2]Raghupathi W, Raghupathi V. An Empirical Study of Chronic Diseases in the United States: A Visual Analytics Approach. Int J Environ Res Public Health. 2018 Mar 1;15(3):431. doi: 10.3390/ijerph15030431. PMID: 29494555; PMCID: PMC5876976. [↑](#footnote-ref-1)
